# Single-cell analysis reveals novel clonally expanded monocytes associated with IL1β–IL1R2 pair in acute inflammatory demyelinating polyneuropathy

**DOI:** 10.1038/s41598-023-32427-5

**Published:** 2023-04-11

**Authors:** Meng Li, Jihe Song, Pengqi Yin, Hongping Chen, Yingju Wang, Chen Xu, Fangchao Jiang, Haining Wang, Baichao Han, Xinshu Du, Wei Wang, Guozhong Li, Di Zhong

**Affiliations:** 1 Department of Neurology, First Affiliated Hospital, Harbin Medical University, Harbin, 150081 Heilongjiang China; 2 Department of Neurology, Heilongjiang Provincial Hospital, Harbin, 150081 Heilongjiang China

**Keywords:** Autoimmunity, Cytokines, Gene regulation in immune cells, Immunological disorders, Neuroimmunology

## Abstract

Guillain–Barré syndrome (GBS) is an autoimmune disorder wherein the composition and gene expression patterns of peripheral blood immune cells change significantly. It is triggered by antigens with similar epitopes to Schwann cells that stimulate a maladaptive immune response against peripheral nerves. However, an atlas for peripheral blood immune cells in patients with GBS has not yet been constructed. This is a monocentric, prospective study. We collected 5 acute inflammatory demyelinating polyneuropathy (AIDP) patients and 3 healthy controls hospitalized in the First Affiliated Hospital of Harbin Medical University from December 2020 to May 2021, 3 AIDP patients were in the peak stage and 2 were in the convalescent stage. We performed single-cell RNA sequencing (scRNA-seq) of peripheral blood mononuclear cells (PBMCs) from these patients. Furthermore, we performed cell clustering, cell annotation, cell–cell communication, differentially expressed genes (DEGs) identification and pseudotime trajectory analysis. Our study identified a novel clonally expanded CD14+ CD163+ monocyte subtype in the peripheral blood of patients with AIDP, and it was enriched in cellular response to IL1 and chemokine signaling pathways. Furthermore, we observed increased IL1β–IL1R2 cell–cell communication between CD14+ and CD16+ monocytes. In short, by analyzing the single-cell landscape of the PBMCs in patients with AIDP we hope to widen our understanding of the composition of peripheral immune cells in patients with GBS and provide a theoretical basis for future studies.

## Introduction

Guillain–Barré syndrome (GBS) is an autoimmune inflammatory peripheral nerve-demyelinating disease^1^. Currently, its pathogenesis is believed to be based on molecular simulations^2^. In other words, exogenous antigens, which have similar epitopes with myelin sheaths, can initiate an adaptive immune response by activating T and B cells in peripheral blood^3^. B cells subsequently produce antibodies that bind to myelin sheath surface antigens. Macrophages then migrate from the blood towards peripheral nerves to phagocytize Schwann cells, damaging the myelin sheath and/or axon in the process^4,5^. In Europe and North America, approximately 90% of GBS cases are of the acute inflammatory demyelinating polyneuropathy (AIDP) subtype^6^. The main pathological features of AIDP involve Schwann cell demyelination, however, axons can be less frequently involved^7^. To date, no antibodies specific to AIDP subtypes have been found in peripheral blood^8^. Moreover, comprehensive changes at the transcription levels of peripheral blood immune cells during peak and recovery periods are poorly understood. In recent years, single-cell transcriptomics has emerged as a revolutionary technology in life sciences studies^9–11^. As an efficient technique, it is widely used to reveal cell heterogeneity and diversity under physiologic and pathologic conditions^12^. Furthermore, studies on GBS have shown that the composition and gene expression of peripheral blood immune cells (PBMCs) in the patients change significantly at the peak stage, which is suitable for single-cell sequencing method^13–17^. Nevertheless, GBS has not been studied by this technique, leaving conceptual and methodological questions on immune process unanswered^18–20^.

Herein, we studied GBS by performing single-cell sequencing on PBMCs from the patients. We extracted PBMCs from three patients with AIDP at the peak stage, two patients with AIDP at the convalescent stage, and three healthy donors. The samples were sequenced and bioinformatically analyzed to construct the first GBS immune cell landscape and to identify disease-related characteristic cell subsets, providing a theoretical basis for future studies.

## Methods

### Patients cohort and clinical characteristics

This study included patients with Guillain–Barre syndrome who were admitted to the First Affiliated Hospital of Harbin Medical University in China between December 2020 and May 2021. The study is a prospective study. According to the NINDS 1990 diagnostic^21^ and electrophysiological criteria^22–25^, five patients with the AIDP subtype were selected. In order to diagnose the patient in combination with symptoms and cerebrospinal fluid results, we selected patients who have no progress in Hughes score for 3 consecutive days from 1 to 2 weeks after the onset of symptoms as typical peak patients. The patients were divided into two groups according to their disease stage; three peak stage (7–14 days after onset) and two late stage (21–30 days after onset). Three HC were selected and matched with respect to sex, and age at the peak stage. Patients with infectious diseases, other demyelinating and autoimmune diseases, as well as using immunomodulatory drugs were excluded.

### PBMCs preparation and single-cell RNA sequencing

PBMCs were isolated from the peripheral blood of patients with AIDP and HC via standard Lymphoprep (Stemcell technologies, Norway) density gradient centrifugation. Samples were cryopreserved in a cryopreservation medium (Sinotech, China), frozen using a slow-freezing process, and stored in liquid nitrogen until use^26^. Isolated cells were counted and diluted to a final concentration of 1100 cells/µL with minimum cell viability of 85%. Single cells were isolated on a chromium controller (BD platform, BD Bioscience) as previously reported^27,28^. Single cells were captured using a BD Rhapsody Single-Cell Analysis System with the BD Rhapsody cDNA Kit, BD Human Sample Multiplexing Kit, BD Rhapsody Cartridge Kit, and BD Rhapsody Cartridge Reagent Kit (BD Biosciences). After cDNA synthesis was completed, RNA-sequencing libraries were constructed using the BD Rhapsody WTA Amplification Kit according to the manufacturer’s instructions (BD Bioscience). Libraries were constructed and sequenced at a depth of approximately 20,000 reads per cell using Illumina Novaseq-6000 (Illumina, San Diego, CA, USA). Data were stored in a public repository (access link: https://202.108.211.75).

### Single cell RNA-seq data processing

Raw sequencing reads of the cDNA library were processed using the BD Rhapsody Whole Transcriptome Assay Analysis Pipeline (v1.1)^29^, which includes filtering by read quality, annotating reads, annotating molecules, determining putative cells, doublets, and generating single-cell expression matrices. The pipeline also determined the sample origin of every single cell via the sample determination algorithm according to the sequencing reads of the SampleTag library. Among all output files, the matrix of UMI counts for each gene per cell was used for downstream analysis. The Genome Reference Consortium Human Build 38 (GRCh38) was used as a reference for the BD pipeline^21^. The output-filtered gene expression matrices were analyzed using R software (v4.1.1) with the Seurat package (4.0.6)^12,30,31^. We read the data of each sample, added the sample source label to each cell, and then integrated all samples including AIDP group and HC group into a Seurat object. After generating the feature-barcode matrix, we selected cells that expressed > 200 genes and 500 UMI in all cells. To exclude low-quality cells from our data, we filtered out cells that expressed mitochondrial genes in > 25% of their total gene expression, as previously described^32^. We integrated datasets normalized with the SCTransform. Next, we performed a principal component analysis (PCA) based on the top 2000 highly variable features. We then performed clustering at a resolution of 0.5 and visualized data using UMAP. Featureplot, Violinplot, and a Heatmap were used to visualize the expression of the indicated genes in each cluster.

### Cell type annotation

We calculated specific markers for each cluster using the FindAllMarkers function with the Wilcoxon test under the following criteria: logfc.threshold = 0.25 and min.pct = 0.1, only.pos = TRUE. To properly identity cell types in filtered samples and combined datasets, we used the R package SingleR (v1.7.1)^33–35^ and manually annotated cell types according to known markers, for example, CD4 + T cells (CD3E, IL7R, CD4, CCR7), CD8 + T cells (CD3E, CD8A, GZMB)^36^, NK cells (GNLY, NKG7, TRAC, TRDC), B cells (MS4A1, CD79A, IGHM)^37^, monocytes (CD14, LYZ, CST3), DC (IL3RA, CLEC4C, NRP1), and platelets (PPBP, TUBB1, PF4)^38–45^ (Supplementary Fig. 3).

### DEGs identification and functional enrichment

Differential gene expression testing was performed using the FindMarkers function in Seurat with parameter ‘test.use = wilcox’ by default, and the DESeq2 method was used to estimate the false discovery rate. DEGs were filtered using min.pct = 0.01 and logfc.threshold = 0.25. Gene set enrichment analysis (GSEA) of Gene Ontology (GO) and Kyoto Encyclopedia of Genes and Genomes (KEGG) terms was performed using the clusterProfiler R package (v4.0.5)^46–48^.

### Cell communication analysis

A systematic analysis of cell communication was based on network analysis and pattern recognition approaches provided by the CellChat (version 1.1.3) R package^49,50^. We used a standard workflow to predict the major signaling inputs and outputs of cells and how these cells and signals coordinate functions. CellChatDB.human was used for the datasets. Subsequently, we classified the signaling pathways and depicted the conserved and context-specific pathways between HC and patients with AIDP.

### Pseudotime trajectory analysis

Developmental trajectories were inferred using Monocle3 (version 1.0.0)^51–54^. A Monocle object is first created according to the expression matrix and metadata information stored in the Seurat object. Single-cell pseudotime analysis for monocyte subsets was performed using Monocle3 with default parameters and the UMAP reduction method. The monocyte cell types were then re-clustered and manually annotated. During feature selection, the top marker genes of the Monocle3 clusters were set as the ordering genes for downstream analysis. Batch effects were eliminated during dimensionality reduction. Trajectory plots and heatmaps were used to show pseudotime results. The expression patterns of representative DEGs were visualized along pseudotime after correcting for estimated size factors and dispersion for all genes.

### Statistical analysis

A two-tailed Wilcoxon rank-sum test was used to compare differences between two elevated groups. *p* < 0.05 was considered significant.

### Ethics approval and consent to participate

The study involving human participants were reviewed and approved by The Ethics Committee of First Affiliated Hospital of Harbin Medical University (No. 2019115). We obtained informed consent from all participants. We confirm that all methods were performed in accordance with the relevant guidelines and regulations.

## Results

### Single-cell transcriptional profiling of PBMCs in AIDP patients

**Figure 1:**
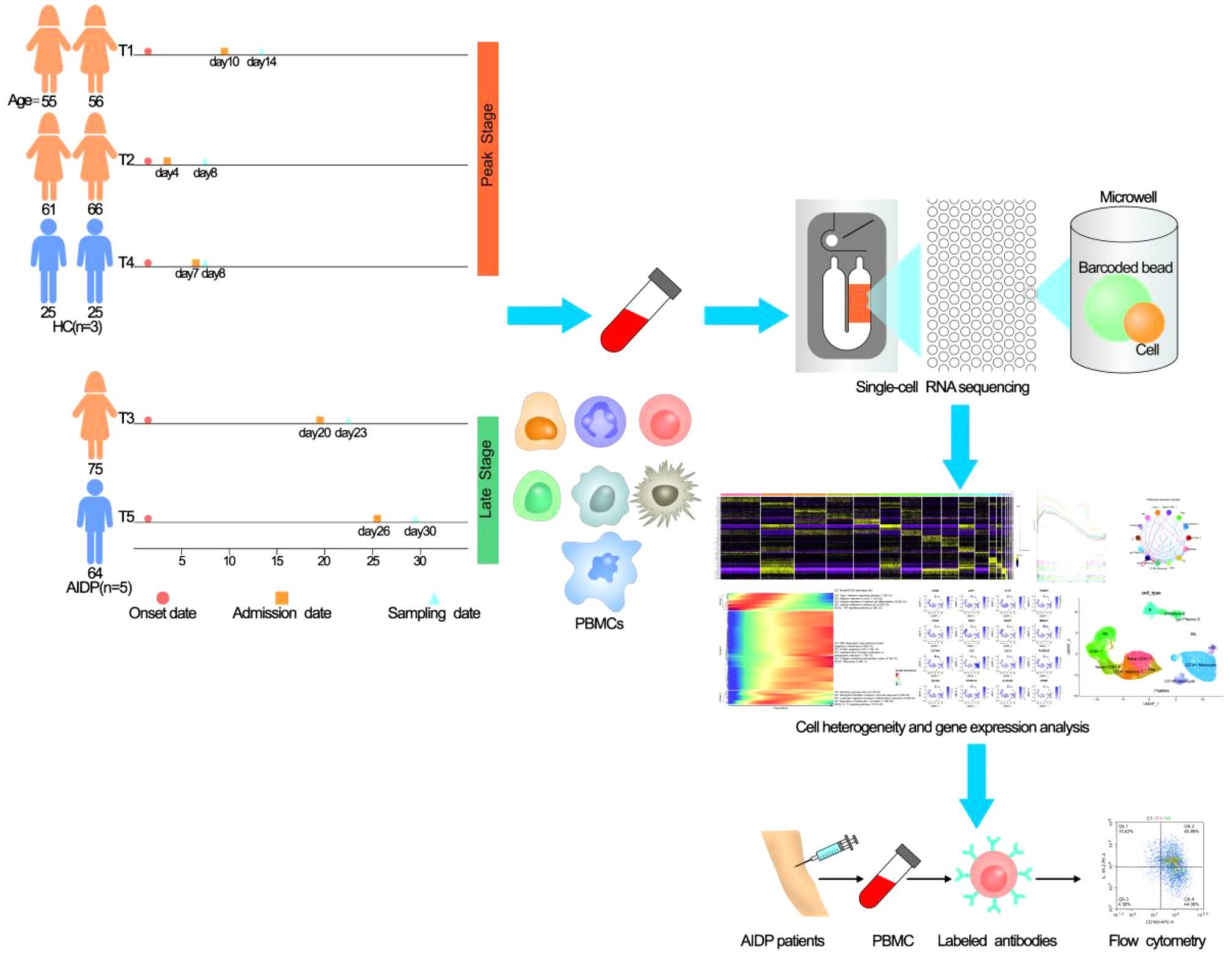
A schematic diagram of the experimental workflow and clinical information of the cohort.

**Figure 2:**
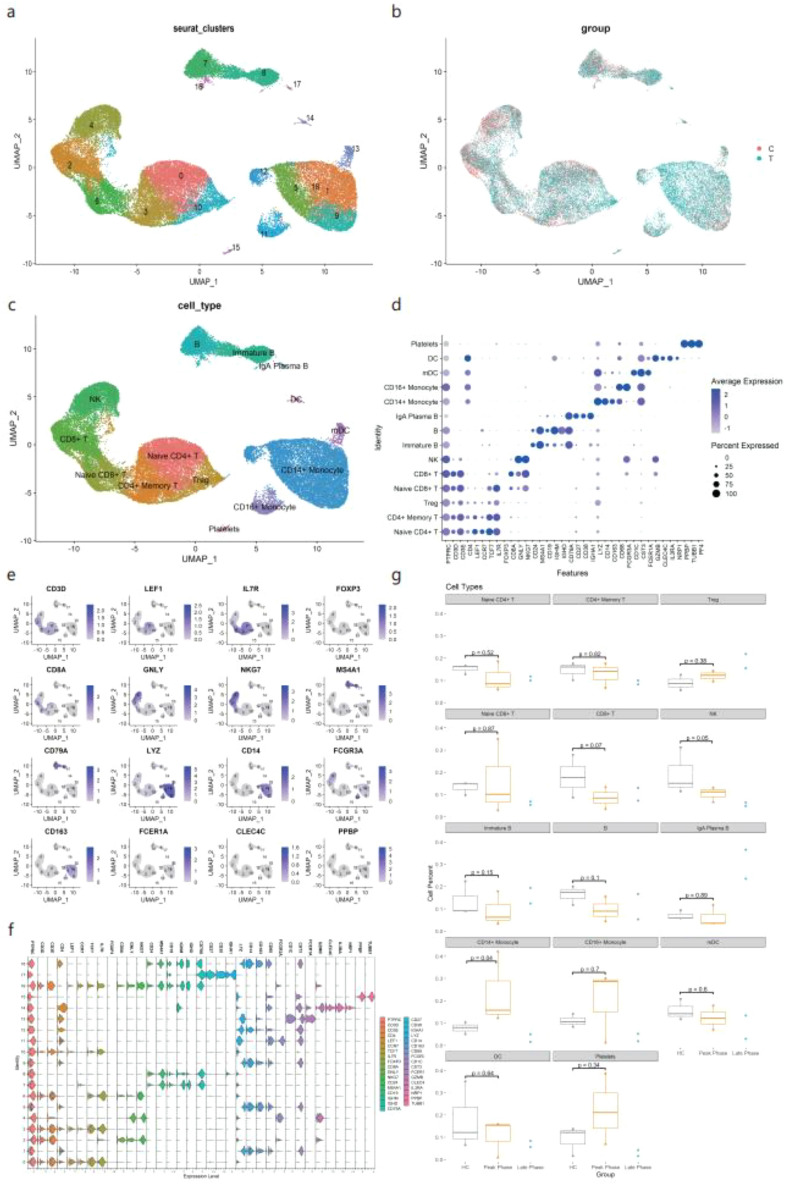
Landscape of PBMCs (peripheral blood mononuclear cells) from HC (healthy control) and AIDP (acute inflammatory demyelinating polyneuropathy) patients. (**a**) A 2D UMAP (uniform manifold approximation and projection) visualization of the identified HC and AIDP patients PBMCs clusters. (**b**) The distribution of HC and AIDP patients derived cells in UMAP, the color of cells are divided by group (C: HC, T: AIDP patients). (**c**) Cell annotation of HC and AIDP patients PBMCs and visualization by UMAP. (**d**) A dot plot shows the expression of reference marker genes in annotated celltypes, the plot color scale represents the average expression and the size scale represents the expression percent of selected marker gene transcripts in each cell type. (**e**) Distribution of classic PBMCs marker genes in each cell of all clusters, the color scale represents expression levels. (**f**) A violin plot is used to display the expression levels of representative PBMCs marker genes across clusters. (**g**) Boxplots show proportions of each cell type in HC and AIDP patients. The x axis correspond to the disease course of each patient. n = 3, n = 3 and n = 2 biologically independent samples for HC, AIDP peak stage and AIDP late stage, respectively. Wilcoxon rank-sum test is used to calculate two-sided P values between HC and AIDP peak stage group, shown all P values. Horizontal lines represent median values, with whiskers extending to the farthest data point within a maximum of 1.5 × interquartile range.

PBMCs extracted from five patients with AIDP (three at the peak stage and two at the late stage) and three healthy controls (HC) were subjected to single-cell RNA sequencing (scRNA-seq); a schematic diagram of the study design is displayed in Fig. 1. The clinical information of the patients with AIDP and HC is shown in Fig. 1 and Supplementary Table 1. After data pre-processing and quality control, 44,002 cells were screened, of which 26,480 cells were from patients with AIDP, and 17,522 cells were from HC. Eighteen clusters were identified and visualized using UMAP for the dimension reduction algorithm (Fig. 2a). To compare the differential cluster distributions between patients with AIDP and HC, cells were separated by group and visualized using UMAP (Fig. 2b). According to the CellMarker database, previous articles, and the SingleR package, all clusters were annotated into 14 cell types. These cell types include naive CD4 + T cells, CD4 + memory T cells, regulatory T (Treg) cells, naive CD8 + T cells, CD8 + T cells, natural killer (NK) cells, immature B cells, B cells, IgA plasma B cells, CD14 + monocytes, CD16 + monocytes, myeloid dendritic cells (mDC), DC, and platelets (Fig. 2c). The reference marker gene expression in annotated cell types is shown in Fig. 2d. The distribution of classic PBMCs marker genes in cells is shown in Fig. 2e, which matched well with the annotated cell types. To compare the differentially expressed genes among clusters, a violin plot was used to display representative marker gene expression levels across clusters (Fig. 2f). The expression levels of most marker genes (CD3D, IL7R, CD8A, MS4A1, IGHA1, CD14, FCGR3A, CD1C, CLEC4C, and PPBP) were specifically expressed in the annotated cell types. Histograms showed a higher proportion of CD14 + monocytes in patients with AIDP than in HC, particularly at the peak stage (Supplementary Fig. S4a). Boxplots were used to display the differences in percentage of annotated cell types across HC, as well as peak and convalescent stage patients (Fig. 2g). Compared to the HC group, the percentage of CD14 + monocytes increased, while the percentage of NK cells decreased in peak stage myeloid dendritic cells patients (Wilcoxon signed-rank test, p < 0.05). To identify the specifically expressed genes in each cluster, a heat map was used to show the top five most differentially expressed genes in the 18 cell clusters (Supplementary Fig. S4b). In general, we clearly distinguished the specific cell types in PBMCs of HC and AIDP patients, and observed an increased proportion of CD14 + monocytes and a decreased proportion of NK cells in patients with AIDP.

### Cell–cell communication patterns in immune cells of AIDP patients

Cell–cell interaction analysis was performed to compare cell–cell communication patterns between patients with AIDP and HC.

**Figure 3:**
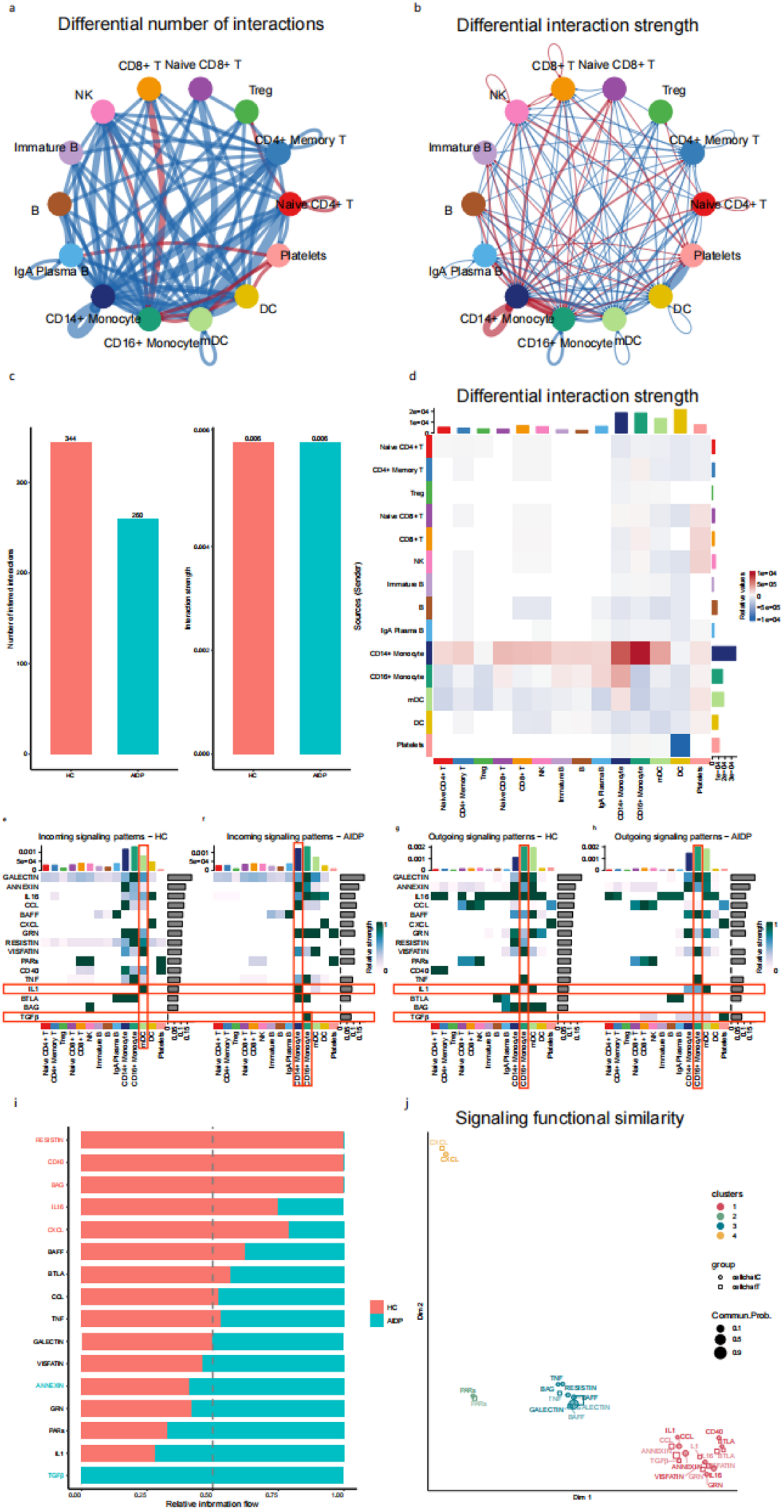
Comparison of cell–cell communication patterns between AIDP patients and HC cell types. (**a**, **b**) Differential number and strength of cell–cell communication between HC (**a**) and AIDP patients (**b**). Compared to HC, the red arrows represent the increased number or strength of interactions in AIDP patients, the blue arrows represent the decreased number or strength of interactions in AIDP patients. The thickness of the arrow represents the number (**a**) or strength (**b**) of interactions between two celltypes. (**c**) Histograms of cell–cell communication number and strength in HC and AIDP patients. (**d**) A heat map of differential cell–cell communication strength between HC and AIDP patients. Red color represents the increased interactions in AIDP patients, blue color represents the decreased interactions in AIDP patients. The top colored bar plot represents the sum of column of values displayed in the heatmap (incoming signaling). The right colored bar plot represents the sum of row of values (outgoing signaling). (**e**–**h**) Heat maps of incoming and outgoing signaling patterns in HC and AIDP patients. The color scale of heat maps indicates relative strength of interactions. The red boxes indicate the significant elevated incoming or outgoing signaling patterns in AIDP patients. The top colored bar plot represents the sum of column of values displayed in the heat maps, the right colored bar plot represents the sum of row of values (signaling pathways). (**i**) The overall information flow comparison of each signaling pathway between HC and AIDP patients. The X axis represents relative information flow, the Y axis represents signaling pathways, the signaling pathways colored red are enriched in HC, and the signaling pathways colored blue were enriched in AIDP patients. (**j**) A 2D visualization of signal functional similarity among all signaling pathways. The shape of the point represents different groups, the color of the point represents different clusters, the size of the point represents communication probability.

‘Interaction number’ means the categories of interaction between two cell types, ‘interaction strength’ means the interaction intensity calculated by gene expression levels between two cell types. The total number of cellular interactions in patients with AIDP decreased compared to HC (Supplementary Fig. S5a,b; Fig. 3c), but the number of cellular interactions between several cell types (CD16 + monocytes and CD8 + T, CD16 + monocytes, NK, IgA plasma B, platelets, etc.) significantly increased (Fig. 3a,c). However, total cellular interaction strength in patients with AIDP did not decrease (Fig. 3b,c), but it rather significantly increased between many cell types (CD14 + monocytes and CD14 + monocytes, CD14 + monocytes and CD16 + monocytes, CD14 + monocytes, NK, CD14 + monocytes, and CD8 + T) (Fig. 3b). Furthermore, the increased interactions were mainly sent from CD14 + monocytes to CD14 + monocytes, CD14 + monocytes to CD16 + monocytes, CD14 + monocytes to mDC, and CD16 + monocytes to CD14 + monocytes. On the other hand, the decreased interactions were mainly sent from platelets to DC (Fig. 3d). The strengths of all incoming and outgoing signaling patterns in each cell type were compared between the HC and patients with AIDP. IL1 signaling was mainly received by mDC in HC; However, in patients with AIDP, it was mainly received by CD14 + monocytes. IL1 signaling was mainly sent from CD14 + monocytes and mDC in HC. In patients with AIDP, the relative strength of outgoing IL1 signaling decreased in CD14 + monocytes and increased in CD16 + monocytes (Fig. 3e–h). TGFβ signaling was absent in HC but significantly increased in patients with AIDP. It was mainly sent from platelets, CD8 + T, NK, IgA plasma B, CD14 + monocytes, CD16 + monocytes, and mDC, however, it was only received by CD16 + monocytes (Fig. 3e–h). A relative information flow analysis was performed to compare the contribution of each signaling pathway between the groups. RESISTIN, CD40, BAG, IL16, and CXCL signaling pathways were mainly constituted by and enriched in cells of the HC group, whereas the TGFβ, IL1, and PARs signaling pathways were mainly constituted by cells of the AIDP group, and TGFβ and ANNEXIN signaling pathways were mainly enriched in cells of the AIDP group (Fig. 3i). All signaling pathways were divided into four clusters according to signaling functional similarity. IL1, TGFβ, GRN, IL16, CD40, ANNEXIN, CCL, VISFATIN, and BTLA signaling pathways all shared functional similarities and composed cluster 1, which had the largest signaling number (Fig. 3j). Moreover, cell–cell communication analysis highlighted attenuated cell–cell interaction abundance but not cell–cell interaction strength between immune cells of patients with AIDP. In patients with AIDP, the specific cell types were mainly CD14 + monocytes and CD16 + monocytes, and the specific pathways focused on several ligand/receptor pairs, such as IL1 and TGFβ.

### AIDP-specific cell types and signaling pathways in the crosstalk of immune cells

**Figure 4:**
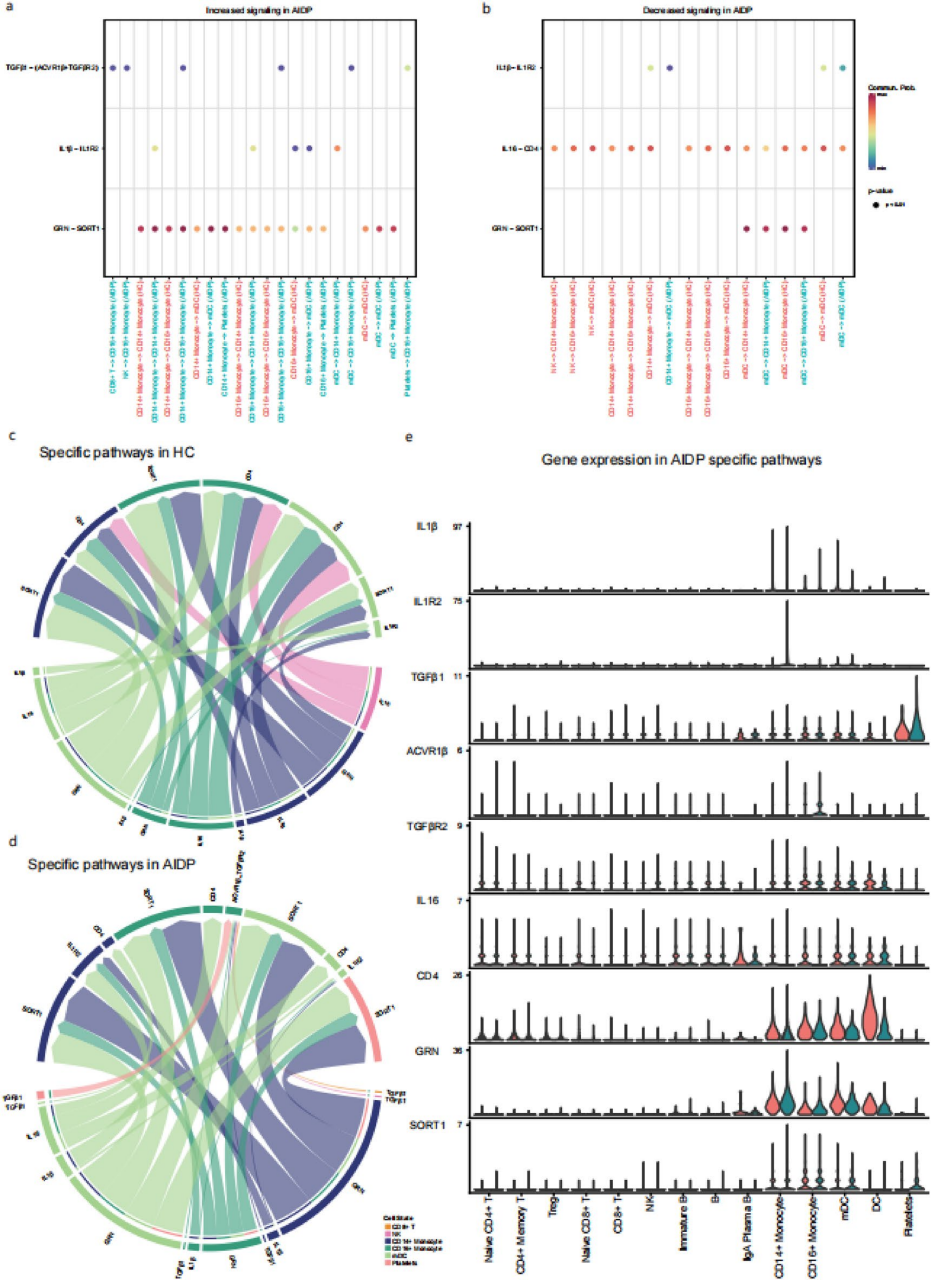
The specific signaling pathways in AIDP patients. a-b. Bubble plot of increased (**a**) and decreased (**b**) signaling in AIDP patients. Signaling and cell types containing AIDP specific are screened as the source and target components. X axis represents the source and target cell types, Y axis represents signaling pathways, the color scale of points represents the communication probability. (**c**, **d**) Chord diagram of signaling ligand-receptor pairs in HC and AIDP patients. Signaling and cell types containing AIDP specific are screened as the source and target components. The color of arrows represents the source cell types, the thickness of arrows represents the proportion of the signaling. (**e**) Gene expression of AIDP specific signaling ligand-receptor pairs between HC and AIDP patients. Violin plots show the distribution of expression levels of each cell type.

Differential incoming and outgoing interaction strength analysis between patients with AIDP and HC was performed to compare the differentially expressed signaling pathways in each cell type. IL1 is an AIDP-specific signaling pathway expressed in CD14 + monocytes (Fig. 3i). Although the strength of IL16 signaling was decreased in the AIDP group (Fig. 3i), it remained an AIDP-specific signaling pathway in Tregs (Supplementary Fig. S5i). ANNEXIN is an AIDP-specific signaling pathway in DC (Supplementary Fig. 5o), whereas GRN is an AIDP-specific signaling pathway in platelets (Supplementary Fig. S5p). Moreover, TGFβ is an AIDP-specific signaling pathway in CD14 + monocytes, CD16 + monocytes, CD8 + T cells, NK cells, plasma B cells, mDC, and platelets (Supplementary Fig. S5c,d,e,f,m,n,p). Regarding AIDP-specific signaling pathways, the IL1β–IL1R2 ligand-receptor pair increased between CD14 + monocytes (source) and CD14 + monocytes (target) as well as between CD16 + monocytes (source) and CD14 + monocytes (target). However, IL1β–IL1R2 mainly decreased between CD14 + monocytes (source) and mDC (target) as well as between mDC (source) and mDC (target). TGFβ1-(ACVR1β + TGFβR2) mainly increased between platelets (source) and CD16 + monocytes (target). GRN-SORT1 mainly increased in the interaction between myeloid-derived cell types (source) and platelet (target) (Fig. 4a). IL16-CD4 mainly decreased in the interaction between NK (source) and myeloid-derived cell types (target) as well as between myeloid-derived cell types (source) and myeloid-derived cell types (target) (Fig. 4b). To compare the input and output signaling patterns between HC and AIDP patients, chord diagrams were used to display the source and target cell types for each signaling pattern. Regarding the IL1β–IL1R2 ligand-receptor pair interaction, CD14 + monocytes switched from being the signaling source to being the target. IL16-CD4 originated from NK and mDC cell types in the HC group, however, it only originated from mDC in the AIDP group. TGFβ 1-(ACVR1β + TGFβR2) was absent in HC, whereas it originated from multiple cell types and targeted CD16 + monocytes in AIDP (Fig. 4c,d). Compared to HC, IL1R2 and GRN expression levels were significantly elevated in CD14 + monocytes, while IL1β and ACVR1β expression levels were mainly elevated in CD16 + monocytes in patients with AIDP. IL16 expression levels were reduced in NK cells, CD14 + monocytes, and CD16 + monocytes, while CD4 expression levels were reduced in mDC and DC. Finally, SORT1 expression was elevated in platelets (Fig. 4e). In summary, after screening several key cell types and signaling pathways, we found that differentially expressed ligand-receptor pairs included IL1β–IL1R2, which originated mainly from CD16 + monocytes and targeted CD14 + monocytes, as well as TGFβ 1-(ACVR1β + TGFβR2), which mainly targeted CD16 + monocytes.

### Landscape of monocytes and characteristics of AIDP patients DEGs in monocyte subsets

**Figure 5:**
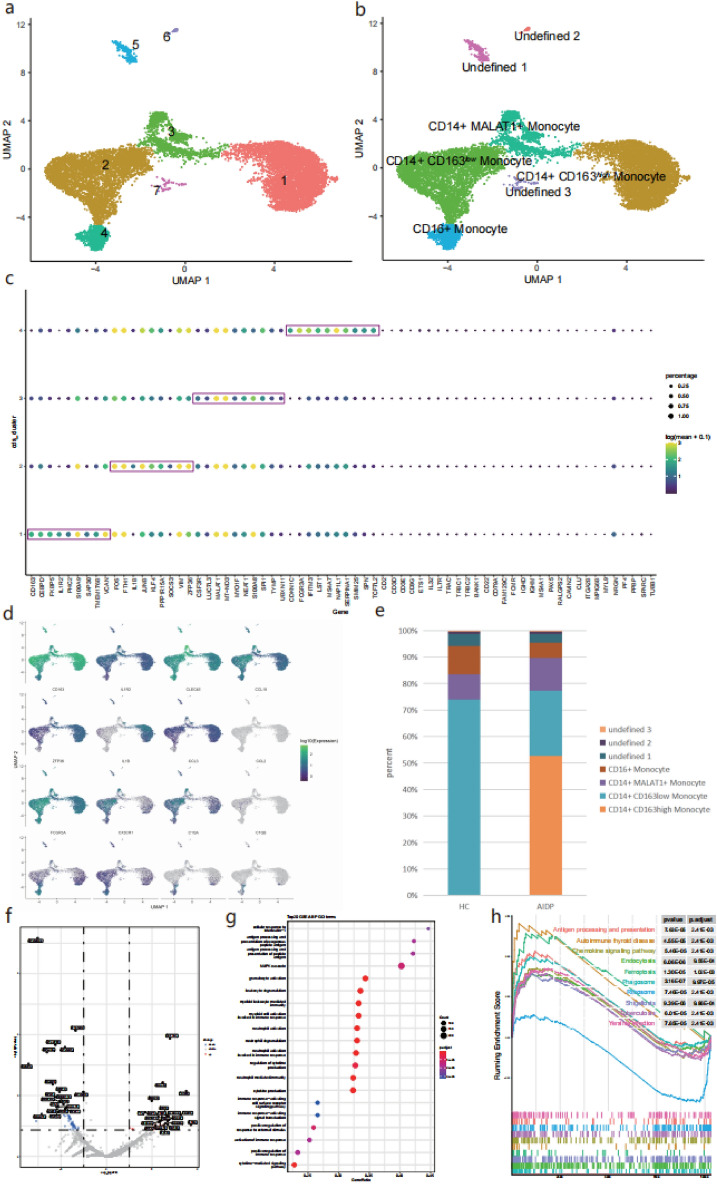
Landscape of monocytes and characteristics of AIDP specific subtype. (**a**) UMAP visualization of cell clusters after re-clustering monocytes. (**b**) UMAP visualization of manually annotated cell types of monocytes. (**c**) A dot plot of top 10 most differentially expressed genes for identified monocyte clusters, the dots of top 10 genes are circled by purple boxes in monocytes clusters. Some genes are overlapped across clusters. Color scale of dots represents the gene expression levels in each cluster, size of dots represents the percentage of cells expressing a gene in each cluster. (**d**) Distribution of some representative marker genes in monocytes. Color scale represents the gene expression levels in each cell. (**e**) Proportion of cell types in each group. The colors indicate cell type information. (**f**) Volcanos of top 20 upregulated and downregulated DEGs between HC and AIDP patients of CD14 + CD163 high monocyte. (**g**) GSEA-GO enrichment analysis in DEGs of CD14 + CD163 high monocyte. Dot plots display top 20 biological process of GO terms according to P value. (**h**) GSEA-KEGG enrichment analysis in DEGs of CD14 + CD163 high monocyte. GSEA enrichment plots display top 10 KEGG pathways according to P value.

To characterize changes in monocyte subsets between HC and patients with AIDP, CD14 + and CD16 + monocytes data obtained from PBMCs of both cohorts were extracted. Monocle3 was used for pre-processing, dimensionality reduction, and clustering. Monocytes were then sub-clustered, and seven clusters were obtained (Fig. 5a). All clusters were annotated according to the expression and distribution of monocyte markers into the following groups: CD14 + CD163 high monocytes, CD14 + CD163 low monocytes, CD14 + MALAT1 + monocytes, CD16 + monocytes, undefined 1, undefined 2, and undefined 3 (Fig. 5b). The top 10 most monocyte-differentially expressed genes (M-DEGs) were then identified in monocyte clusters and certain genes overlapped across clusters. The macrophage-related genes CD163 and CEBPD were CD14 + CD163 high monocyte-specific M-DEGs. The FOS family-related genes FOS and FOSB were CD14 + CD163 low monocyte-specific M-DEGs. Interestingly, the IL1 pathway-related receptor IL1R2 was a CD14 + CD163 high monocyte-specific M-DEG, whereas the IL1 pathway-related ligand IL1β was a CD14 + CD163 low monocyte-specific M-DEG. Also, certain CD14 + MALAT1 + monocyte-specific M-DEGs were conserved, such as MALAT1 and NEAT1. CD16 + monocyte-specific M-DEGs were FCGR3A, IFITM3, and LST1 (Fig. 5c). Representative marker genes distribution in each monocyte is shown in Fig. 5d. The complement system-related genes C1QA and C1QB were distributed in CD16 + monocytes and CD14 + CD163 high monocytes. Some macrophage-related genes, including CD163, CEBPD, CLEC4E, CCL18, and IL1R2, were distributed in CD14 + CD163 high monocytes. Several chemokines, including CCL2, CCL3, and IL1β, were mainly distributed in CD14 + CD163 low monocytes, and the chemokine receptor CX3CR1 was mainly distributed in CD16 + monocytes. The proportion of each cell type in each group was subsequently calculated (Fig. 5e). CD14 + CD163 high monocytes were not abundant in the healthy controls, however their proportion in the patients with AIDP was more than 50%. The proportion of CD14 + CD163 low monocytes was greater than 70% in HC, but it was significantly reduced in patients with AIDP. DEGs between HC and patients with AIDP in each cell type were compared. A total of 96 DEGs were screened in CD14 + CD163 high monocytes; and EEF1A1, HLA-C, CD163, and IL1R2 were found to be elevated in patients with AIDP (Fig. 5f). In addition, antigen processing and presentation, the MAPK cascade, and some myeloid cell-related immune responses were also reported (Fig. 5g). Furthermore, CD14 + CD163 high monocyte DEGs were enriched in chemokine signaling pathways, antigen processing and presentation, autoimmune thyroid disease. 9 out of the 10 top KEGG pathways were upregulated (Fig. 5h). 55 DEGs were screened in CD14 + CD163 low monocytes, MTRNR2L8, IFITM3, and IFI6 were elevated in patients with AIDP, while HLA-DRB5 and LYZ were decreased in patients with AIDP (Supplementary Fig. S7a). CD14 + CD163 low monocyte DEGs were enriched in SRP-dependent co-translational proteins that target the membrane, proteins that target ER biological processes and osteoclast differentiation, and the TNF signaling pathways (Supplementary Fig. S7b,c). 17 DEGs were screened in CD14 + MALAT1 + monocytes, and CD163 and IL1R2 were found to be elevated in AIDP patients (Supplementary Fig. S7d). CD14 + MALAT1 + monocyte DEGs were enriched in mitochondrial ATP synthesis-coupled electron transport and some neurodegenerative disease-related pathways. 16 DEGs were screened in CD16 + monocytes, MTRNR2L8 and IFITM3 were elevated in AIDP patients, while HLA-DRB5 and RPS26 were decreased in AIDP patients (Supplementary Fig. S7g). CD16 + monocyte DEGs were enriched in negative regulators of viral processes, certain cellular homeostasis-related biological processes, and antigen processing and presentation pathways. Notably, seven out of the 10 top KEGG pathways were downregulated (Supplementary Fig. S7h,i). Collectively, we identified a significantly increased CD14 + CD163 high-monocyte subtype in AIDP patients, which highly expressed IL1R2, and a decreased CD14 + CD163 low-monocyte subtype in AIDP patients which highly expressed IL1β. Furthermore, DEGs of CD14 + CD163 high monocytes in patients with AIDP were significantly enriched in the cellular responders to interleukin- 1 and chemokine signaling pathways,indicating that CD14 + CD163 high monocytes may migrate out of blood vessels under the action of chemokines, and the IL1 signaling pathway may activate these monocytes during the AIDP inflammatory process.

### Monocytes trajectories and key DEGs vary over pseudotime in monocytes

**Figure 6:**
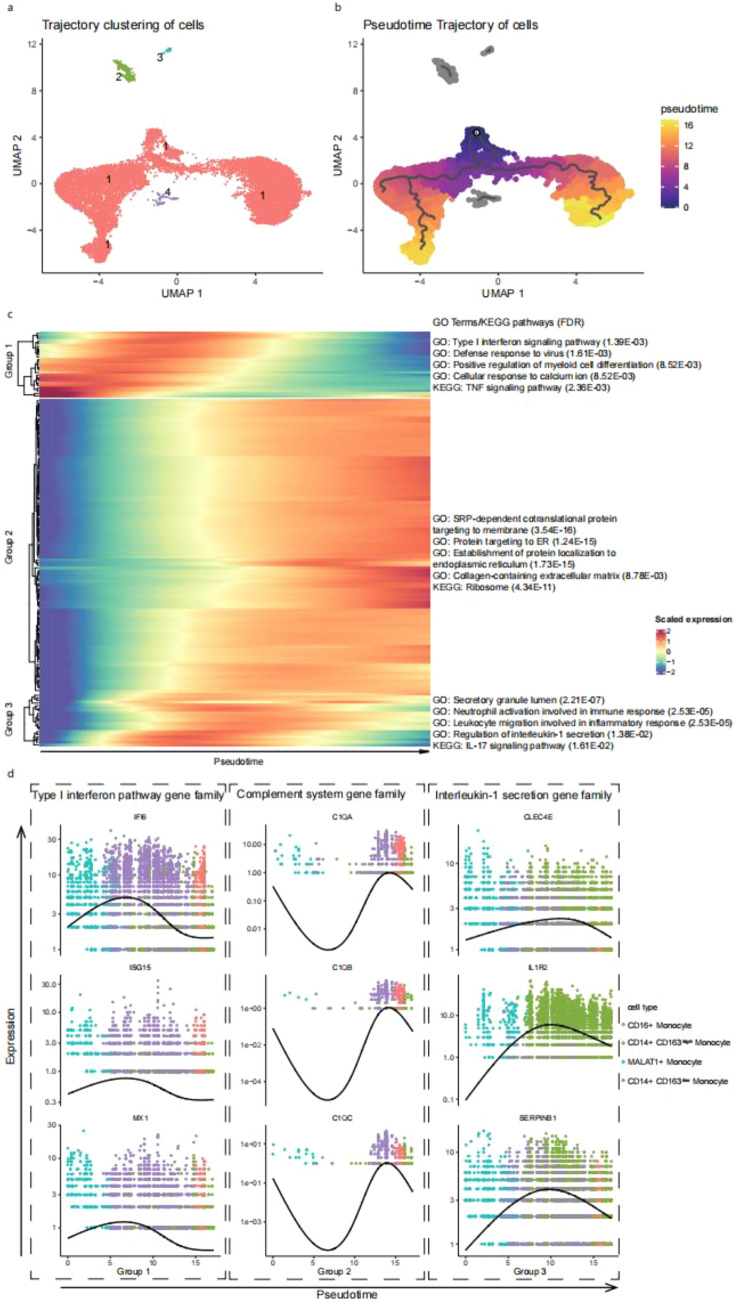
Pseudotime trajectory analysis of monocytes. (**a**) Trajectory clustering of cells. The color represents the different trajectories. (**b**) Pseudotime trajectory of cells. The node represents the starting point of monocytes. The color scale represents the pseudotime progression. The line represents the trajectories of differentiation. (**c**) Heatmap representing the transcriptomic progression of dynamic DEGs in the AIDP patients monocytes through the pseudotime. DEGs are from the comparison of HC and AIDP patients in 4 monocyte clusters. DEGs were grouped by expression profiles with k-means. The heatmap shows the normalized average Z score of each group. Associated GO terms, KEGG pathways and false discovery rates (FDR) are reported for each group, the ComplexHeatmap R package (2.8.0) was used to generate the heatmap (https://github.com/jokergoo/ComplexHeatmap). (**d**) Select protein families and pathways that are differentially expressed across pseudotime corresponding to gene groups in (**c**). Dot coloring corresponds to cell types of monocytes. Curves are fitted for expression along pseudotime.

To construct the potential differentiation trajectories of monocytes and better understand the progression between cellular states, all monocytes were computationally ordered along pseudotime. First, a slingshot algorithm was used to reconstruct monocytes into four trajectories (Fig. 6a). Trajectory 1 contained most cells and consisted of four monocyte clusters (Fig. 5b). To further clarify the progression between the clusters, CD14 + MALAT1 + monocytes were defined as initiator cells to determine the starting point of monocyte differentiation. They are terminally differentiated in two directions: CD14 + CD163 high monocytes and CD16 + monocytes. CD14 + MALAT1 + monocytes also went through an intermediate phase of CD14 + CD163 low monocytes during their differentiation into CD16 + monocytes (Fig. 6b). A total of 157 DEGs between HC and AIDP patients in four monocyte clusters were extracted and grouped according to their expression profiles using k-means. Three broad patterns were identified. A heatmap was used to represent transcriptomic progression of dynamic DEGs in AIDP monocytes along pseudotime. It showed the normalized average Z score of each gene, and the associated GO terms, KEGG pathways, and false discovery rates for each group (Fig. 6c). The expression levels of group 1 DEGs increased at the early stage and decreased at the late stage of the monocyte trajectory. They were enriched in the type 1 interferon signaling pathway and regulate myeloid cell differentiation and TNF signaling pathway. The Type 1 interferon family genes were elevated early during the trajectory, highlighting their roles at the early stage of AIDP (Fig. 6d). Group 2 DEGs expression levels were decreased at the early stage and gradually increased at the late stage. They are enriched in SRP-dependent co-translational proteins that target the membrane, proteins that target the ER, and ribosomes. In addition, complement system family gene expression levels were elevated late during the trajectory and displayed a consistent trend (Fig. 6d). Group 3 DEGs expression levels were low at the early stage, rose during the metaphase stage, and fell back to normal at the late stage. They were enriched in the secretory granule lumen, regulation of interleukin-1 secretion and the IL-17 signaling pathway. Interleukin-1 secretion family gene expression levels were elevated during the metaphase stage, implicating their participation in the AIDP inflammatory process during the peak stage (Fig. 6d).

### Characteristics of T cell subsets in patients with AIDP

**Figure 7:**
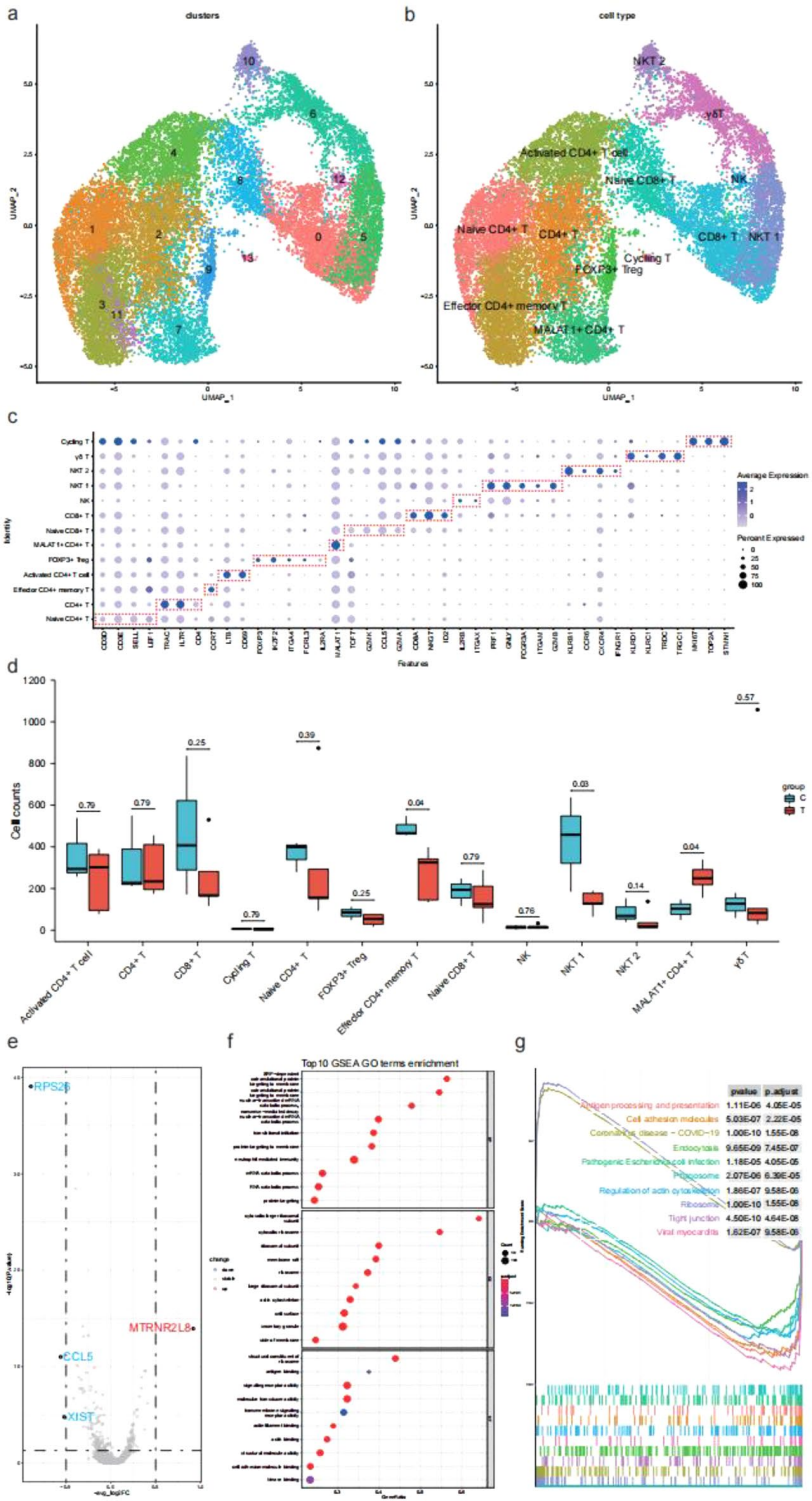
Landscape and characteristics of T cell subsets. (**a**) UMAP visualization of HC and AIDP patients T cell clusters. The color represents different clusters. (**b**) Cell annotation of HC and AIDP patients T cell clusters and visualization by UMAP. (**c**) A dot plot shows the expression of reference marker genes in T cell types, the plot color scale represents the average expression and the size scale represents the expression percent of selected marker gene transcripts in each cell type. The dots of each cell type specific genes are circled by red boxes. (**d**) Boxplots show proportions of each T cell type in HC and AIDP patients. The x axis correspond to cell types, the y axis correspond to cell counts. Wilcoxon rank-sum test is used to calculate two-sided P values between HC and AIDP patients, shown all P values. Horizontal lines represent median values, with whiskers extending to the farthest data point within a maximum of 1.5 × interquartile range. (**e**) A volcano of upregulated and downregulated DEGs between HC and AIDP patients in MALAT1 + CD4 + T cells. (**f**) GSEA-GO enrichment analysis in DEGs of MALAT1 + CD4 + T cells. A dot plot displays top 10 BP, CC and MF of GO terms according to adjust P value. (**g**) GSEA-KEGG enrichment analysis in DEGs of MALAT1 + CD4 + T cells. A GSEA enrichment plot displays top 10 KEGG pathways according to adjust P value.

To characterize changes in individual T cell subsets between HC and AIDP patients, T cells from PBMCs were sub-clustered into 14 clusters (Fig. 7a). Thirteen cell types identified based on canonical T-cell markers expression and distribution were as follows: six CD4 + T cells subtypes (naive CD4 + T, CD4 + T, effector CD4 + memory T, activated CD4 + T, FOXP3 + Treg, MALAT1 + CD4 + T), two CD8 + T cells subtypes (naive CD8 + T, CD8 + T), two NKT cells subtypes (NKT 1, NKT 2), one γ6T cells subtype, one cycling T cells subtype, and one NK cells subtype (Fig. 7b). Among the CD4 + T subtypes clusters, we identified effector CD4 + memory T cells expressing high CCR7 levels, activated CD4 + T cells expressing high levels of LTB and CD69, FOXP3 Treg cells (FOXP3 + , IKZF2 + , ITGA4 + , FCRL3 + , and IL2RA +), and MALAT1 + CD4 + T cells expressing high MALAT1 levels (Fig. 7c). Among the two NKT subtypes clusters, NKT 1 cells expressed high levels of PRF1, GNLY, FCGR3A, ITGAM, and GZMB, while NKT 2 cells expressed high levels of KLRB1, CCR6, CXCR4, and IFNGR1. Moreover, we identified a γ6T cell type (KLRC1+, KLRD1+, TRDC, and TRGC1) and a cycling T cell type (MKI67+, TOP2A+, and STMN1). To obtain T cell subset characteristics of patients with AIDP, we analyzed the distribution of each cell subtype between the two groups. Notably, in the CD4 + T cell subtypes of patients with AIDP, the proportion of effector CD4 + memory T cells decreased, whereas that of MALAT1 + CD4 + T cells significantly increased (Fig. 7d). NKT1 and NKT2 showed downward trends in NKT cell subtypes, wherein NKT1 proportion was significantly decreased in patients with AIDP. To further investigate differential transcriptomic genes in elevated cell types of AIDP patients, we compared the expression profiles of MALAT1 + CD4 + T cells between HC and AIDP patients. We observed that the pseudogene MTRNR2L8 was upregulated, whereas the ribosomal genes RPS26, CCL5, and XIST were downregulated in AIDP patients (Fig. 7e). We observed that DEGs between HC and AIDP patients were enriched in certain biological processes including SRP-dependent co-translational proteins targeting the membrane, cytosolic large ribosomal subunit, and structural constituents of the ribosome (Fig. 7f). In the top 10 enriched KEGG pathways, cell adhesion molecules and endocytosis were mainly downregulated, while COVID-19 coronavirus disease and ribosomes were upregulated (Fig. 7g). In summary, we identified a significantly increased MALAT1 + CD4 + T cell subtype, and the DEGs of this subtype were enriched in certain ribosome-related processes.

### Features of NK cell subsets in AIDP patients

To identify the features of the NK cell subsets, we divided NK cells into 11 clusters (Supplementary Fig. S9a). We then annotated each cluster into 11 cell types according to the expression of NK cell markers and highly expressed genes (Supplementary Fig. S9b–d). Five subtypes of NK (ZAFT high NK, FCER1G high NK, CCL5 high NK, MALAT1 NK, and FOS high NK) and four subtypes of NKT (IL32 high NKT, FGFBP2 NKT, GNLY NKT, and IL7R NKT) were identified. One subtype of pro-NK cells expressed high levels of SELL, TCF7, and LEF1, and one subtype of cycling NK cells expressed high MKI67, TOP2A, and STMN1 levels (Supplementary Fig. S9c). Next, we compared the proportion of each cell type in the HC and AIDP groups. Most mature NK and NKT cell types showed a downward trend, however, the difference was not statistically significant, except for ZAFT-high NK cells (p < 0.05) (Supplementary Fig. S9e). Hence, we performed differential gene expression and gene enrichment analyses of the ZAFT-high NK subtype. MALAT1 was upregulated, whereas RPS26 and GNLY were downregulated in the AIDP group compared to the HC group (Supplementary Fig. S9f). Notably, the proportion of ZAFT-high NK cells decreased in the AIDP group, however, the top 10 GO terms of ZAFT-high NK DEGs between the HC and AIDP groups were all upregulated and enriched in cellular response to type I interferon (IFN-I) as well as mRNA splicing biological processes (Supplementary Fig. S9g). Likewise, the top 10 KEGG pathways were all upregulated, and were mainly enriched in CDVID-19 and IL-17 immune-related signaling pathways (Supplementary Fig. S9h). Collectively, most NK subtypes showed a downward trend in AIDP patients, and the ZAFT-high NK subtype showed a significant reduction. Moreover, ZAFT-high NK DEGs in AIDP patients were mainly enriched in IFN-I and other immune-related pathways.

### Features of specific plasma subtypes in AIDP patients

To investigate the characteristics of the B cell subsets, we subclustered B cells into 10 clusters and annotated them into 10 cell types according to the expression of B cell markers and highly expressed genes (Supplementary Fig. S10a,b). Five subtypes of B cells (transitional B1, transitional B2, immature B, S100A10 high B, and TPT1 high B cells), two subtypes of plasma cells (plasma and IgA plasma), pDC, NKT, and platelets were identified. Of the two plasma subtypes, the plasma cluster expressed high levels of CD38, CD27, CD81, NFKBIA, and immunoglobulin family genes, and the top three expressed genes were S100A4, XBP1, and HSP90B1. Plasma IgA specifically expressed IGHA1, IGHA2, and JCHAIN (Supplementary Fig. S10c,d).

The proportion of each B subtype, including plasma and IgA plasma, was not significantly different between the HC and AIDP groups (Supplementary Fig. S10e). Furthermore, differential gene expression and gene enrichment analyses showed that some mitochondrial genes (MT-ND2, MT-ATP8, and MT-ND4L) were upregulated in plasma cells of the AIDP group, and the top 10 BP GO terms and KEGG pathways of plasma DEGs were mainly enriched in clathrin-coated endocytic vesicle membranes, MHC class II protein complexes, and cell adhesion molecules (Supplementary Fig. S10f–h). IGHM were upregulated in IgA plasma cells, and the top 10 BP GO terms and KEGG pathways of plasma DEGs were mainly enriched in spliceosomes as well as mRNA splicing via spliceosome and transesterification reactions (Supplementary Fig. S10i–k). Overall, we identified five B subtypes and two plasma subtypes in the PBMCs of patients with AIDP, with no significant differences in proportion. However, the gene expression profiles of the plasma and IgA plasma cells in patients with AIDP showed significant changes.

## Discussion

AIDP is an autoimmune mediated-peripheral nerve demyelinating disease^55^, and its pathogenesis likely involves molecular simulation^56^. Certain exogenous antigens share epitopes with Schwann cells that can stimulate immune cells to generate a misguided immune response^57^ causing specific macrophages to migrate to the peripheral nerves and damage Schwann cells^58^. Previous studies have focused on classical molecular biology and animal models, and thus obtaining comprehensive scenarios of cellular and molecular immune responses during AIDP becomes difficult. To address this issue, we built an immune landscape in PBMCs of patients with AIDP at single-cell resolution, depicted the dynamic nature of cellular responses, and identified immune cell types that play important roles in AIDP pathogenesis.

We identified 13 common immune cell types including T cells, B cells, DC, and myeloid cells, in patients with AIDP and HC according to the authoritative markers of peripheral blood immune cells as described in previous studies^38,59^. Among them, the proportion of CD14 + monocytes was significantly elevated during the peak stage of AIDP and decreased at the late stage. A retrospective clinical study was conducted in 114 patients with GBS and 120 healthy controls. The results showed that the absolute monocyte count was significantly elevated in patients with GBS, and the monocyte count had significant positive correlations with disease severity of GBS^60^. Our results were consistent with this study. Therefore, at the early stage of the immune response, CD14 + monocytes may proliferate and differentiate after being stimulated by immune signals, and then subsequently migrate out of the blood vessel and damage peripheral nerves. This mechanism is consistent with previous studies that showed that macrophages damage Schwann cells through phagocytosis^4,58,61,62^. On the other hand, NK cells decrease significantly at the peak stage of AIDP and continue to decline in the late stage. A previous study obtained peripheral blood samples of 20 patients with GBS within 7 days after onset of neuropathic symptoms, the results showed that NK cell activity in patients with GBS was significantly decreased compared with that in 20 control subjects^63^. Our results were consistent with this study as well. This indicates that NK cell reduction may be involved in the development and immune imbalance of AIDP.

In terms of cell communication, we found that the number of interactions between immune cells, but not their strength, decreases during the acute stage of PBMCs from AIDP patients. Based on these findings, we believe that cell interactions shift from diversification to simplification during AIDP, which allows rapid activation of the immune response. Next, the differential interaction pathways between HC and patients with AIDP were compared. Our results revealed that the differential interaction strength between CD14 + monocytes and other immune cell types increased, especially between CD14 + and CD16 + monocytes and within CD14 + monocytes. This indicates that certain communication processes between monocytes increase in AIDP, which may play an important role in the acute immune response.

Regarding specific signaling pathways, AIDP increased the relative flow of information through the IL1 and TGFβ pathways compared to HC. The IL1 pathway, which is mainly a CD14 + monocyte-receiving and a CD16 + monocyte-delivering pathway, was increased, suggesting that the interaction between CD16 + monocytes (as the source) and CD14 + monocytes (as the target) may be involved in AIDP pathogenesis. Simultaneously, the TGFβ signaling pathway increased in various immune cells in AIDP, which indicates that this signaling pathway might also be activated in a variety of immune cells of AIDP. The similar signaling pathway functions of IL1 and TGFβ implied the presence of a synergistic effect in the disease process. Then, we screened AIDP-specific receptor-ligand pairs. Compared to HC, we found that IL1β—the source ligand of the IL1β–IL1R2 pair—exhibited increased expression in CD16 + monocytes in AIDP, while IL1R2—the target receptor—exhibited increased expression in CD14 + monocytes. On the other hand, the TGFβ1-(ACVR1β + TGFβR2) ligand-receptor pair mainly targeted CD16 + monocytes in AIDP. On the other hand, the TGFβ1-(ACVR1β + TGFβR2) ligand-receptor pair mainly targeted CD16 + monocytes in AIDP.

Among monocyte subsets, CD14 + CD163 high monocytes were significantly elevated and highly expressed IL1R2 in patients with AIDP. In addition, their DEGs were enriched in the IL1 and chemokine signaling pathways. On the other hand, CD14 + CD163 low monocytes and CD16 + monocytes greatly expressed IL1β. A previous study demonstrated that monocytes were the predominant IL1β-producing cell population in the peripheral blood of neonatal-onset multisystem inflammatory disease (NOMID) patients^64^. This study and our results suggested that monocytes were the main source of IL1β signaling in autoimmune diseases. Currently, it is believed that IL1 receptors are mainly divided into two categories: IL1R1 and IL1R2. Previous studies suggested that IL1R1 is the receptor for IL1, and IL1R2 is suggested to be a decoy receptor, because it lacks the signal-transducing TIR domain in the cytoplasmic part^65,66^. Meanwhile, a study comfirmed that in acute myocardial infarction (AMI), the expression of IL1R2 in M2 type macrophages was elevated, which was a potential biomarker of AMI^67^. Therefore, the roles of IL1R2 in health and disease remain largely unknown.

**Figure 8:**
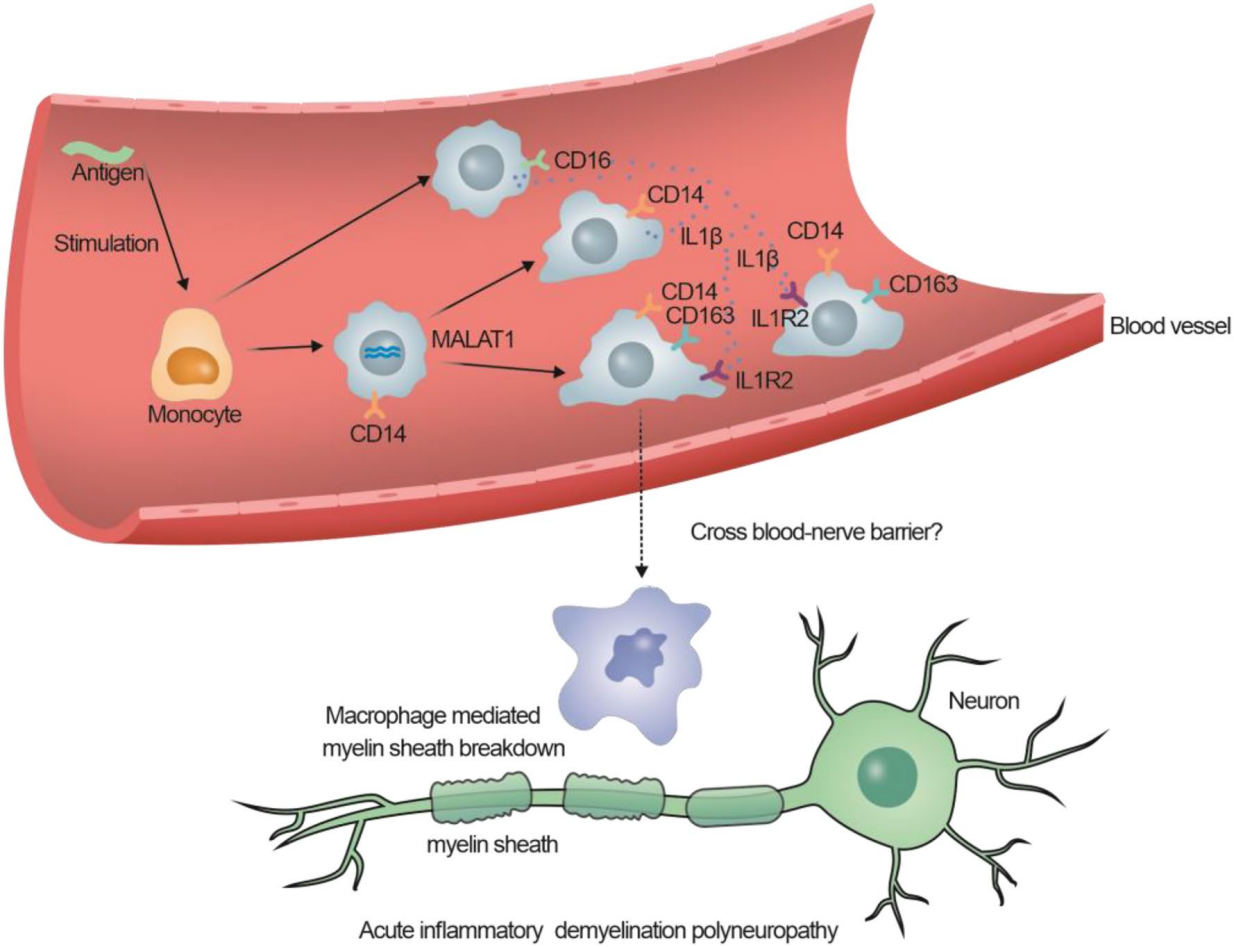
The proportion of CD14 + CD163 + cells in AIDP patients and the potential mechanism of monocytes in AIDP.

Previous analysis of cellular communication showed that communication through the IL1 pathway between CD14 + monocytes and CD16 + monocytes and within CD14 + monocytes was enhanced in patients with AIDP. Therefore, we speculate that during the acute phase of AIDP, the CD14 + CD163 + IL1R2 + monocyte cell types, which receive IL1β signals from CD14 + and CD16 + monocytes, may be amplified in peripheral blood and subsequently migrate to peripheral nerves under the action of chemokine pathways, leading to macrophage mediated phagocytosis or peripheral nerve demyelination (Fig. 8).

Furthermore, after pseudotime trajectory analysis of monocytes, we found that monocytes originated from CD14 + MALAT1 + monocytes and were divided into two branches. This suggested that monocytes of patients with AIDP differentiated in two directions: CD14 + CD163 high monocytes and CD16 + monocytes. The DEGs of monocytes in patients with AIDP and HC were sorted and clustered according to time. We found that the interferon 1 pathway gene family increased during the early stage and decreased during the late stage of AIDP in peripheral blood, suggesting that interferon 1 pathway-related genes may play a role in the early stage of the disease. Similarly, the complement system gene family may play a role in the late stage and the IL1 secretion gene family may play a role in the peak stage. However, their specific trends and mechanisms of action require further validation.

Among the T cell subsets, only MALAT1 + CD4 + T cells showed a significant increase whereas the proportion of most cell subsets of AIDP did not change significantly compared to HC. MALAT1 + CD4 + T cell DEGs were enriched in ribosome-related pathways, suggesting that there may be a class of CD4 + T cells that highly express MALAT1, which plays a role in AIDP through ribosomal pathways. Among the NK cell subsets, most clusters showed a downward trend in AIDP, with the ZAFT-high NK subset decreasing significantly. However, the type I IFN-related pathways were significantly upregulated in the ZAFT-high NK subset, suggesting that the type I IFN pathway may play an important role in the NK subset of AIDP.

This study had limitations that need to be addressed. First, the scRNA-seq analysis sample size was limited, which affected the statistical power of differential expression and abundance analyses. Second, different disease stages corresponded to different patients in this study, which may have increased cohort heterogeneity. Third, some patients diagnosed with AIDP were treated with IVIG, which might affect the innate and adaptive immune responses. In addition, we performed scRNA analysis on PBMCs of patients with AIDP, which may not completely reflect the local immune responses in the peripheral nerve myelin sheath. Therefore, longitudinal studies are required to uncover the characteristics of different immune cell types, and further experimental studies are needed to validate the immunological changes in AIDP. In addition, because the mRNA expression level and protein level of a gene may not be completely consistent, if we jointly analyze the GBS single-cell transcriptome data and proteomic data, and find consistent results at the gene expression level and protein level, it may have more potential clinical significance. However, at present, the proteomic or other omics data from GBS patient samples are particularly rare, so our research only analyzes the composition of peripheral blood immune cells and the cell–cell communication at the single-cell resolution. If more GBS proteomic data appear in the future, we will perform a multi-omics analysis to find more potential biomarkers and therapeutic targets.

## Conclusions

To the best of our knowledge, this is the first study to build the PBMCs landscape of patients with AIDP at single-cell resolution. Furthermore, this study provides a new perspective to better understand the immunological responses in the peripheral blood of AIDP and guides future efforts for developing drugs that specifically target AIDP.

## Supplementary Information

Supplementary Legends.

Supplementary Figures.

Supplementary Information 3.

Supplementary Information 4.

Supplementary Information 5.

Supplementary Information 6.

Supplementary Information 7.

Supplementary Information 8.

## Data Availability

The raw sequencing data, expression-count data are deposited at NODE with the project ID: OEP002315 and OEP002701 (https://www.biosino.org/node/). Technical scRNA-seq information and data tables with details of the included patients are included as Supplementary Tables.

## Abbreviations

GBS: Guillain–Barré syndrome
PBMCs: Peripheral blood mononuclear cells
AIDP: Acute inflammatory demyelinating polyneuropathy
HC: Healthy controls
scRNA-seq: Single-cell RNA sequencing
Treg: Regulatory T
NK: Natural killer
mDC: Myeloid dendritic cells
M-DEGs: Monocyte-differentially expressed genes
IFN-I: Type I interferon
GRCh38: Genome reference consortium human build 38
PCA: Principal component analysis
UMAP: Uniform manifold approximation and projection
GSEA: Gene set enrichment analysis
GO: Gene ontology
KEGG: Kyoto encyclopedia of genes and genomes
