# Supplementary material for: Single-cell analysis reveals novel clonally expanded monocytes associated with IL1β–IL1R2 pair in acute inflammatory demyelinating polyneuropathy
Source: Sci Rep. 2023 Apr 11;13:5862. doi: 10.1038/s41598-023-32427-5 (PMC10088807; doi:10.1038/s41598-023-32427-5)
Supplement: Supplementary file 1 — Supplementary Legends. [file 41598_2023_32427_MOESM1_ESM.docx]

Supplementary table legend:

Supplementary table 1. The clinical characteristics of the patients with AIDP and HC.

Supplementary table 2. GSEA analysis data of monocyte subsets.

Supplementary table 3. GSEA analysis data of MALAT1+ CD4+ T subsets.

Supplementary table 4. GSEA analysis data of ZAFT high NK subsets.

Supplementary table 5. GSEA analysis data of plasma and IgA plasma subsets.

Supplementary table 6. Technical scRNA-seq information with details of the included patients.
