# Supplementary material for: Single-cell analysis reveals novel clonally expanded monocytes associated with IL1β–IL1R2 pair in acute inflammatory demyelinating polyneuropathy
Source: Sci Rep. 2023 Apr 11;13:5862. doi: 10.1038/s41598-023-32427-5 (PMC10088807; doi:10.1038/s41598-023-32427-5)
Supplement: Supplementary file 2 — Supplementary Figures. [file 41598_2023_32427_MOESM2_ESM.docx]

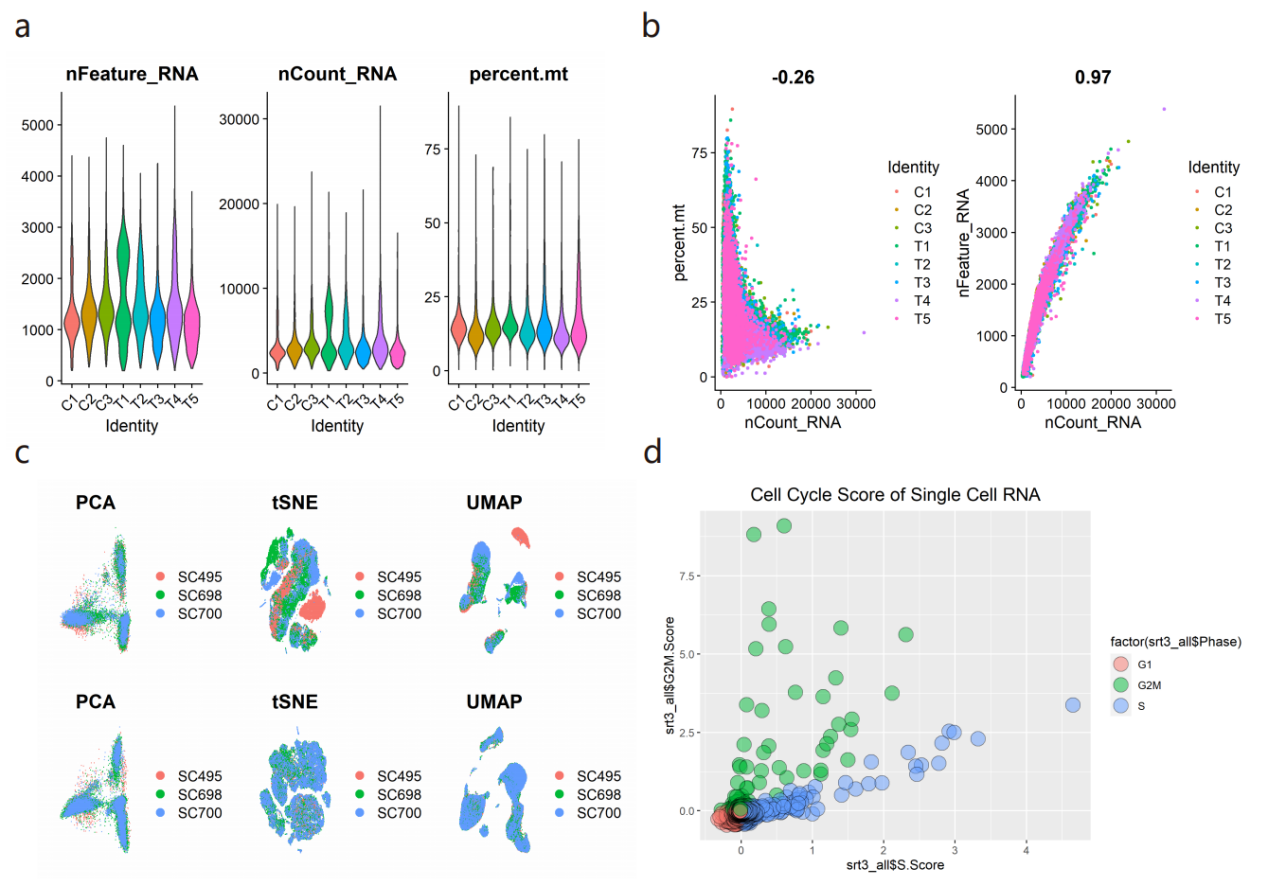


**Supplementary figure 1.** Quality control and data filtering. a. An violin plot of the number of RNA features, the number of RNA counts, and the percentage of mitochondria in each cell of each sample. b. The correlation of the number of RNA counts and the percentage of mitochondria, and the number of RNA features in each sample. c. PCA, t-SNE, and UMAP methods were used to cluster the merged degree of each chip derived cell before and after eliminating batch effects on the data with the SCT method. Colors represented batches. d. Fraction of cell cycles for each cell were calculated, with the x-axis representing the S score and the y-axis representing the G2M score.


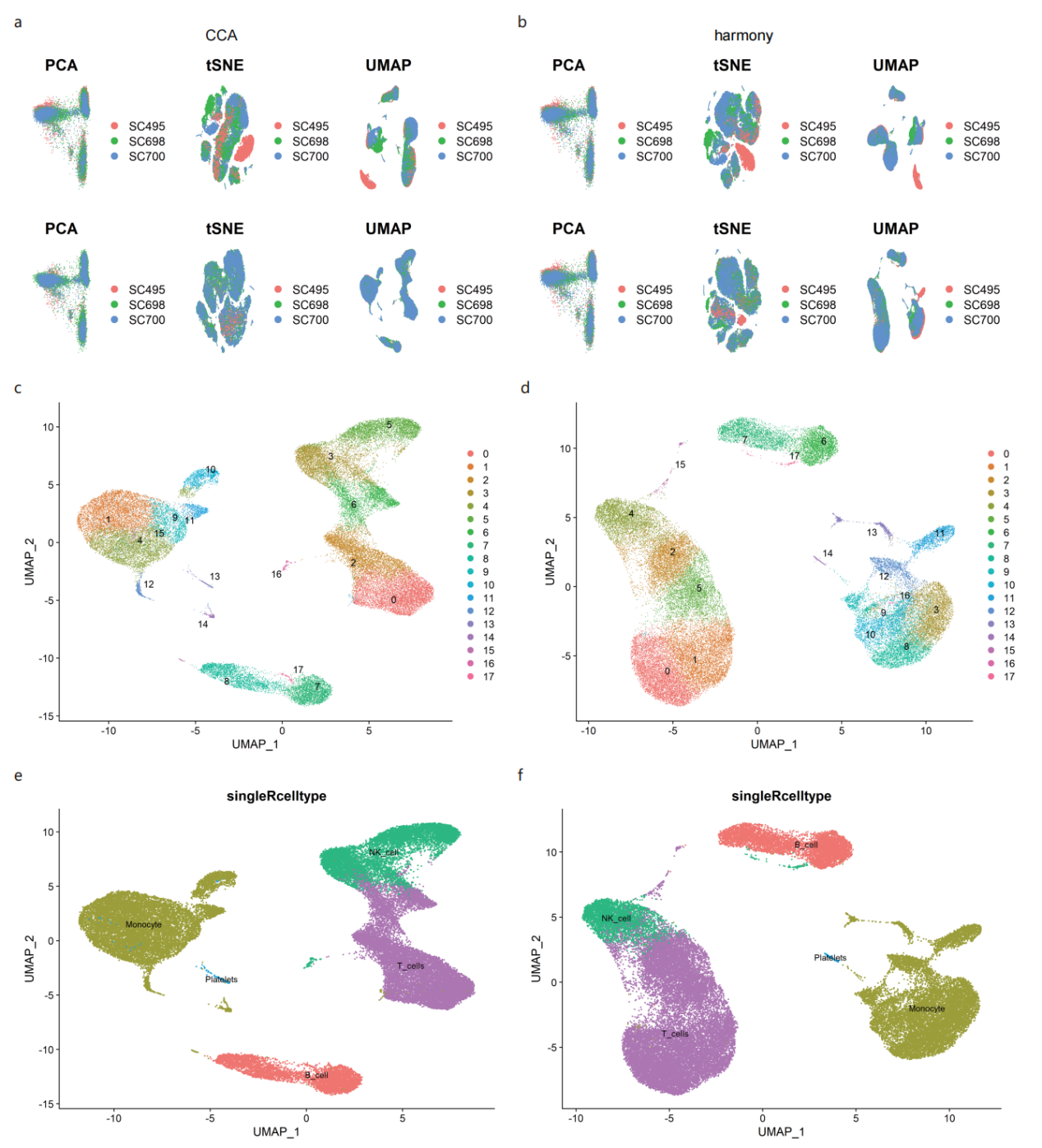


**Supplementary figure 2**. CCA and the harmony methods to eliminate batch effects. a-b. (a) CCA and (b) harmony PCA, t-SNE and UMAP visualization before and after eliminating batch effects, with dots representing cells and color representing batches. c-d. UMAP visualization of cell clusters after eliminating batch effects with (c) CCA and (d) harmony methods, we choose a resolution of 0.5. e-f. UMAP visualization of cell annotation by SingleR package after eliminating batch effects with (c) CCA and (d) harmony methods.


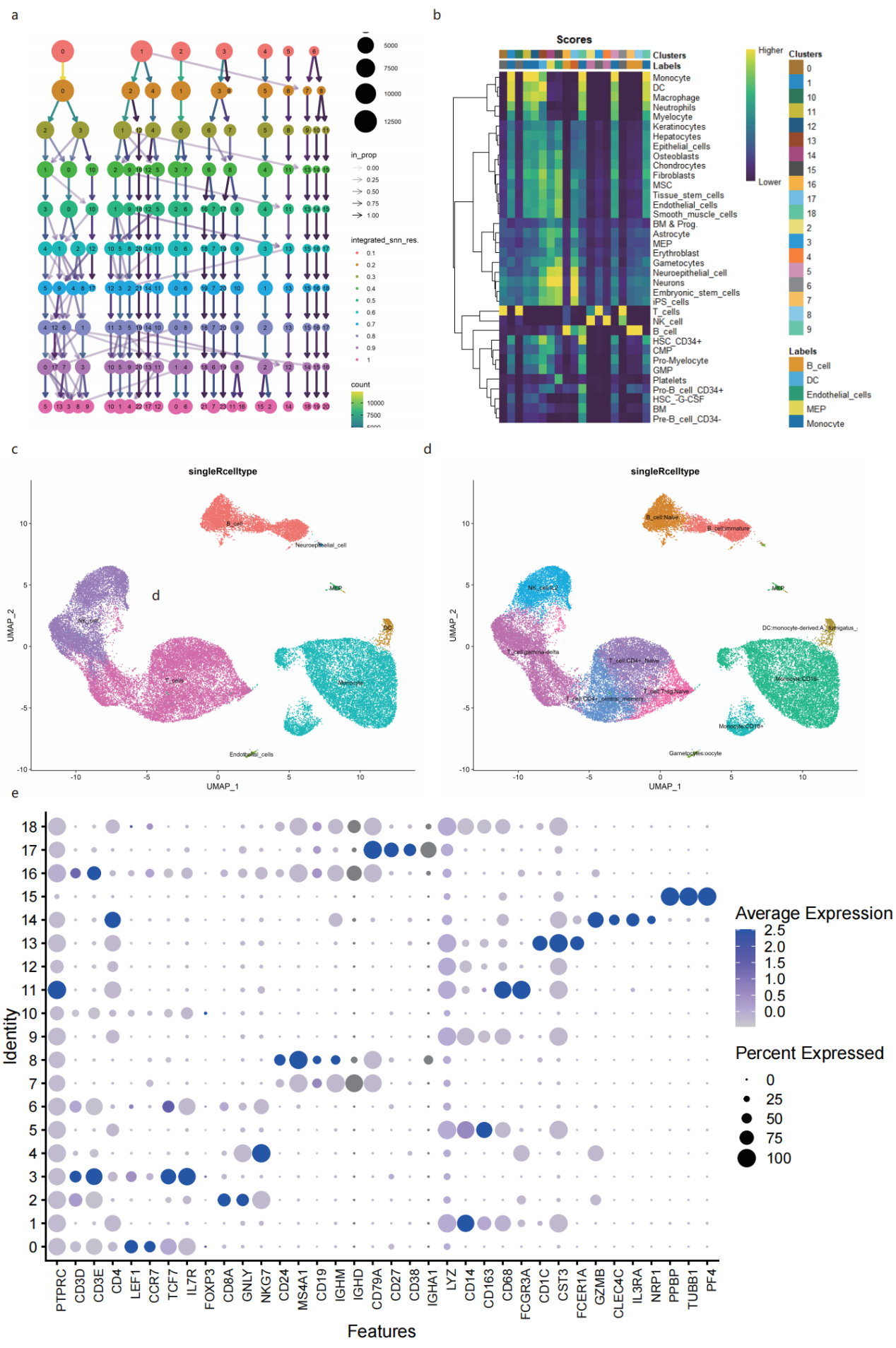


**Supplementary figure 3**. Reference results for cell clustering and annotation. a. Clustree plot of cell clustering under different resolution parameters. b. Heatmap of individual cluster to cell type correlations, annotated according to SingleR main reference set, the SingleR R package (1.7.1) was used to generate the heatmap (https://github.com/LTLA/SingleR). c,d. Cell type annotation according to the two reference datasets of SingleR (c) main and (d) fine. e. A dotplot of immune cell typical markers expressed in each cluster, x axis represents genes, y axis represents clusters, scale of circle represents expressed percent and color of circle represents average expression.


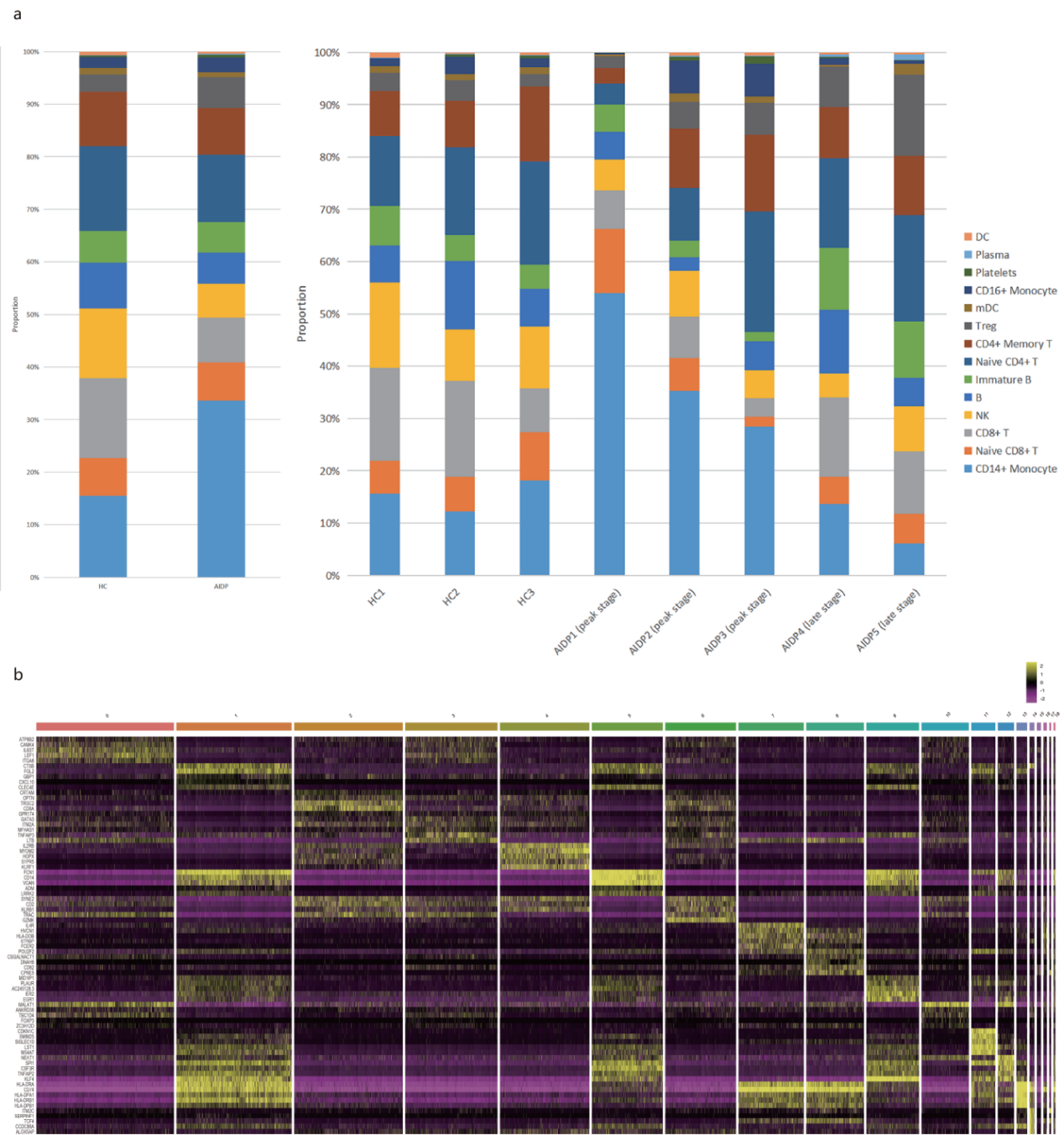


**Supplementary material 4**. Proportion and differentially expressed genes of each cell type. a. Proportion of cell types in each group and each sample. The colors indicate cell type information. b. A heat map based on the top 5 most differentially expressed genes for each cell subset in all 18 clusters, the Seurat R package (4.0.6) was used to generate the heatmap (https://satijalab.org/seurat/index.html).


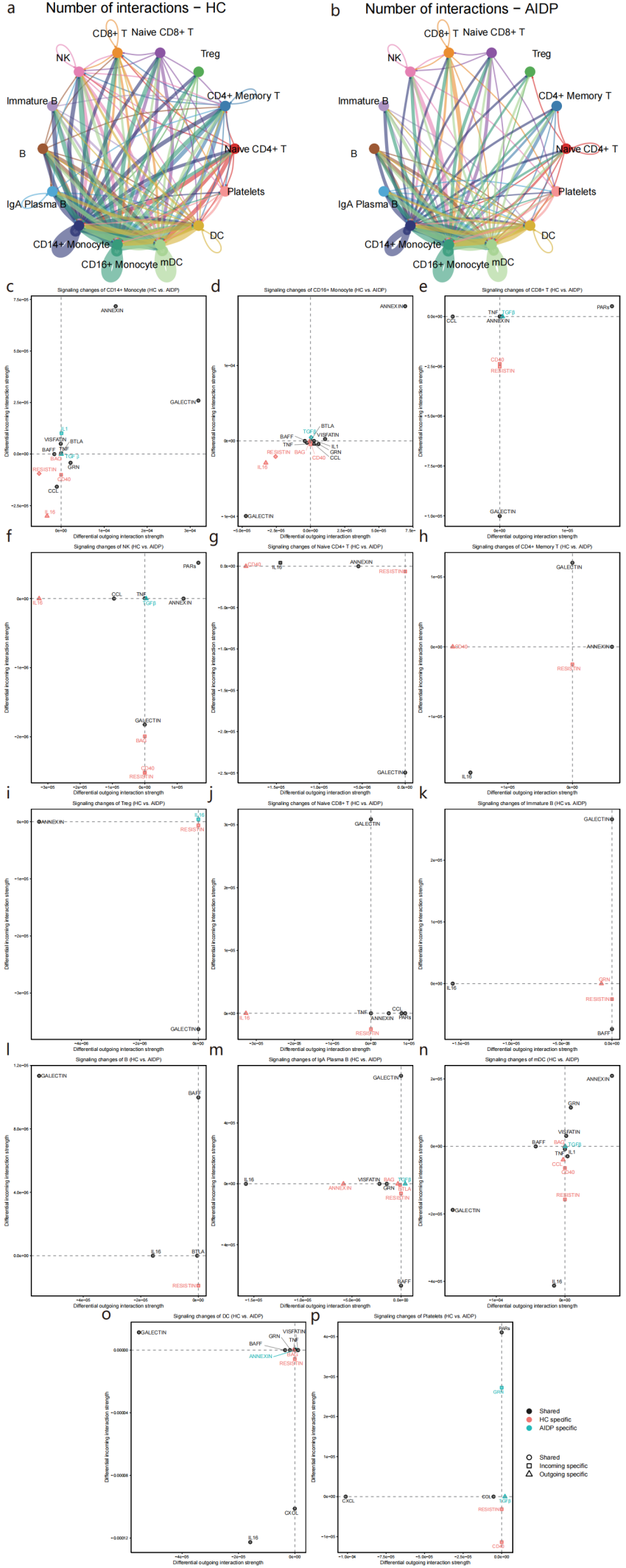


**Supplementary material** **5**. Cell-cell communication patterns and the specific signaling pathways of each cell type in AIDP patients. a-b. Number of cell-cell communication in HC (a) and AIDP patients (b). The colors of arrows indicate the cell type which is the source of interactions between two cell types, the thickness of arrows indicate number of interactions between two cell types. c-p. In each cell type, differentially expressed signaling pathways between AIDP patients and HC. The black, red or blue color respectively represent shared, HC specific or AIDP specific signaling pathways, the circle, rectangle or triangle shape respectively represent shared, incoming specific or outgoing specific signaling pathways.


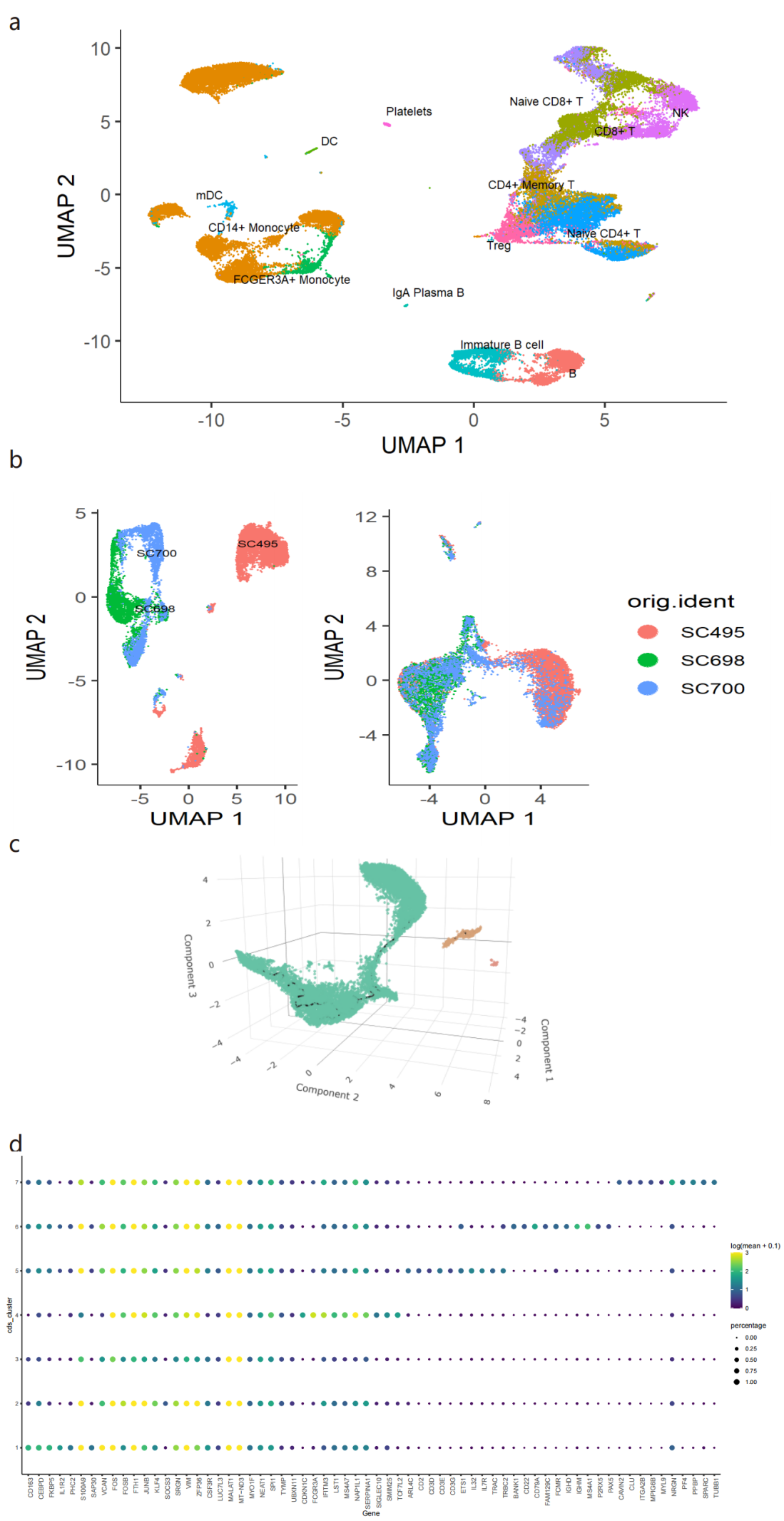


**Supplementary material** **6**. Quality control and clustering of monocytes. a. Mapping of annotated monocytes on UMAP visualization after re-dimensionality reduction and re-clustering of all cells using Monocle3. b. UMAP visualization of monocyte subsets before and after eliminating of batch effects. Color represents cells from different batches. c. 3D diagram of monocyte trajectory. Different colors represent different trajectories. d. A dot plot of top 10 most differentially expressed genes for each cell cluster, the dots of top 10 genes are circled by purple boxes in monocytes clusters. Some genes are overlapped across clusters. Color scale of dots represents the gene expression levels in each cluster, size of dots represents the percentage of cells expressing a gene in each cluster.


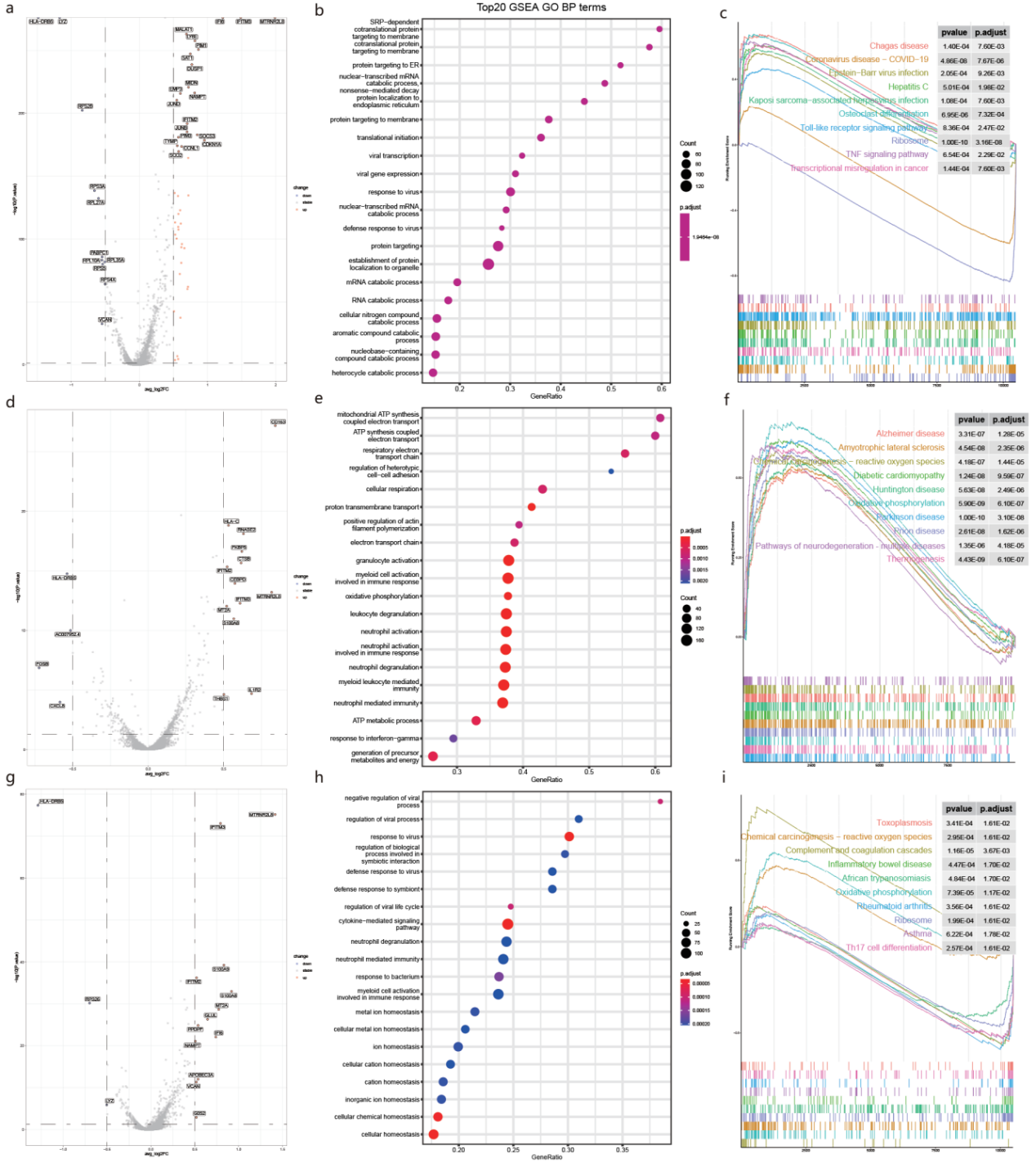


**Supplementary material** **7.** Characteristics of other monocyte subtypes. a,d,g. Volcanos of top 20 upregulated and downregulated DEGs between HC and AIDP patients of 3 monocyte subtypes. b,e,h. GSEA-GO enrichment analysis in DEGs of 3 monocyte subtypes. Dot plots display top 20 biological process of GO terms according to P value. c,f,i. GSEA-KEGG enrichment analysis in DEGs of 3 monocyte subtypes. GSEA enrichment plots display top 10 KEGG pathways according to P value.


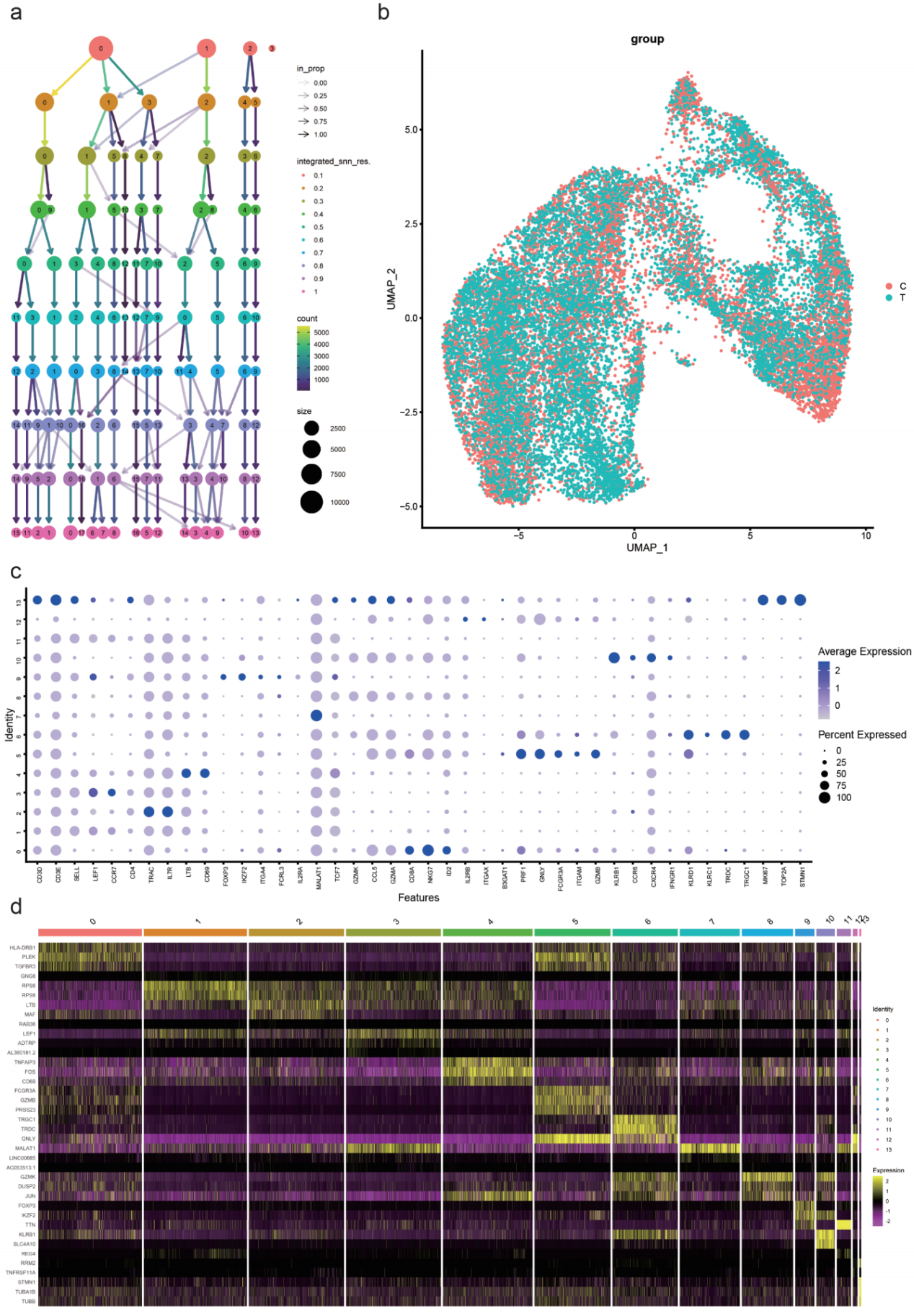


**Supplementary material** **8**. Reference results for T cell subsets clustering and annotation. a. A clustree plot of T cells clustering under different resolution parameters. b. The distribution of HC and AIDP patients derived T cells in UMAP, the color of cells are divided by group (C: HC, T: AIDP patients). c. A dotplot of immune cell typical markers expressed in each T cell cluster, x axis represents genes, y axis represents clusters, scale of circle represents expressed percent and color of circle represents average expression. d. A heat map based on the top 3 most differentially expressed genes in all 14 clusters.


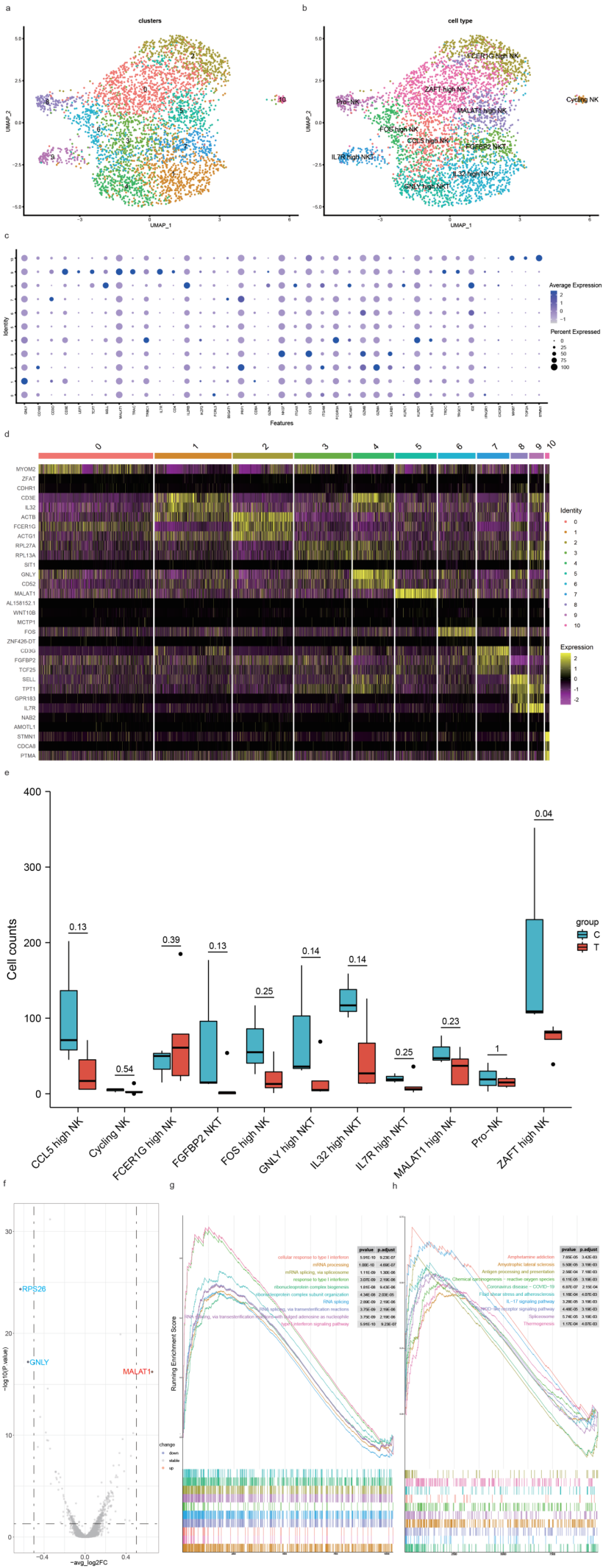


**Supplementary material 9**. Landscape and characteristics of NK subsets.

1. UMAP visualization of HC and AIDP patients NK cell clusters. The color represents different clusters.
2. Cell annotation of HC and AIDP patients NK cell clusters and visualization by UMAP.
3. A dot plot shows the expression of reference marker genes in NK cell clusters, the plot color scale represents the average expression and the size scale represents the expression percent of selected marker gene transcripts in each cluster.
4. A heat map based on the top 3 most differentially expressed genes for each cell subset in all 11 clusters.
5. Boxplots show proportions of each NK cell type in HC and AIDP patients. The x axis correspond to 11 cell types, the y axis correspond to cell counts. Wilcoxon rank-sum test is used to calculate two-sided P values between HC and AIDP patients, shown all P values. Horizontal lines represent median values, with whiskers extending to the farthest data point within a maximum of 1.5 × interquartile range.
6. A volcano of upregulated and downregulated DEGs between HC and AIDP patients in ZAFT high NK cells.
7. GSEA-GO enrichment analysis in DEGs of ZAFT high NK cells. A GSEA enrichment plot displays top 10 biological process of GO terms according to adjust P value.
8. GSEA-KEGG enrichment analysis in DEGs of ZAFT high NK cells. A GSEA enrichment plot displays top 10 KEGG pathways according to adjust P value.


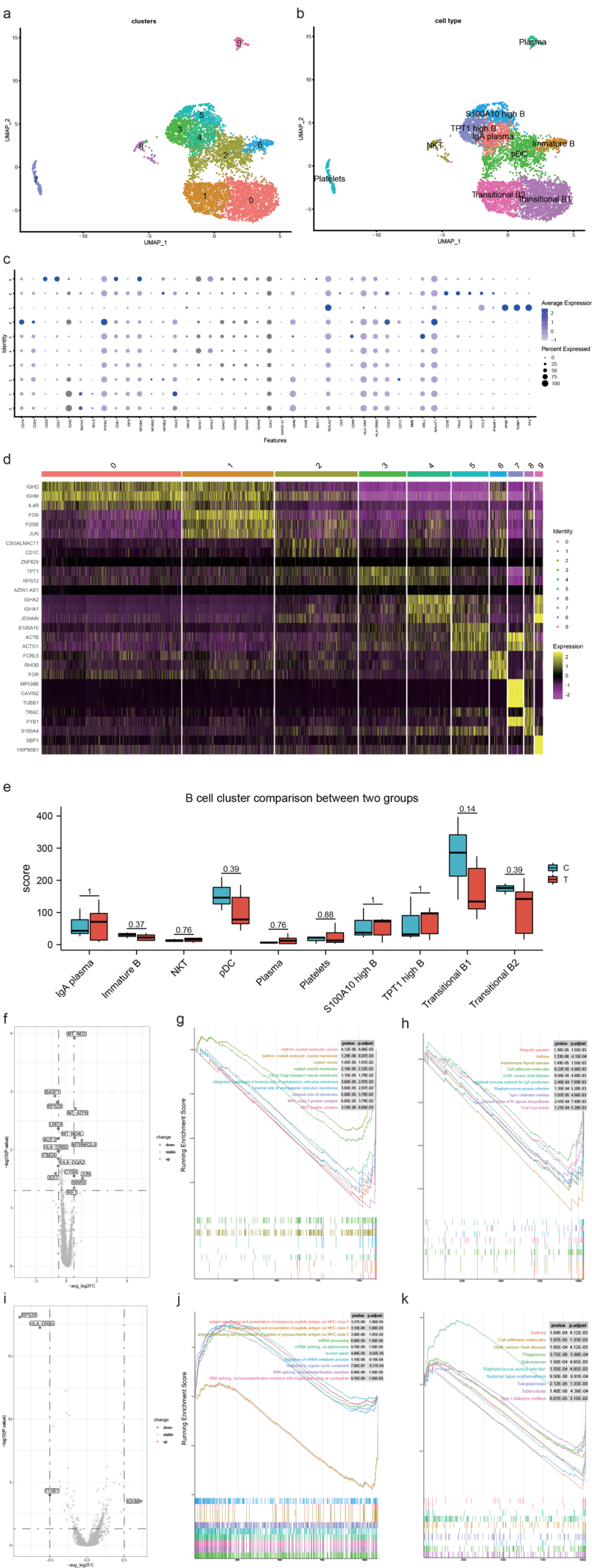


**Supplementary material 10.** B cell atlas and features of specific B subtypes. a. UMAP visualization of HC and AIDP patients B cell clusters. The color represents different clusters. b. Cell annotation of HC and AIDP patients B cell clusters and visualization by UMAP. c. A dot plot shows the expression of reference marker genes in B cell clusters, the plot color scale represents the average expression and the size scale represents the expression percent of selected marker gene transcripts in each cluster. d. A heat map based on the top 3 most differentially expressed genes for each B cell cluster. e. Boxplots show proportions of each B subtype in HC and AIDP patients. The x axis correspond to 11 subtypes, the y axis correspond to cell counts. Wilcoxon rank-sum test is used to calculate two-sided P values between HC and AIDP patients, shown all P values. Horizontal lines represent median values, with whiskers extending to the farthest data point within a maximum of 1.5 × interquartile range. f. Volcanos of upregulated and downregulated DEGs between HC and AIDP patients in plasma and IgA plasma, respectively. g. GSEA-GO enrichment analysis in DEGs of plasma and IgA plasma. GSEA enrichment plots display top 10 biological process of GO terms according to adjust P value. h. GSEA-KEGG enrichment analysis in DEGs of plasma and IgA plasma. GSEA enrichment plots display top 10 KEGG pathways according to adjust P value.
